# Systematic Review of Gender-Specific Child and Adolescent Mental Health Care

**DOI:** 10.1007/s10578-023-01506-z

**Published:** 2023-02-27

**Authors:** Lena Herrmann, Franziska Reiss, Inga Becker-Hebly, Christiane Baldus, Martha Gilbert, Gertraud Stadler, Anne Kaman, Lina Graumann, Ulrike Ravens-Sieberer

**Affiliations:** 1 Department of Child and Adolescent Psychiatry, Psychotherapy, and Psychosomatics, University Medical Center Hamburg-Eppendorf, Martinistraße 52, W29, 20246 Hamburg, Germany; 2 German Center for Addiction Research in Childhood and Adolescence, University Medical Center Hamburg-Eppendorf, Hamburg, Germany; 3 Institute of Gender in Medicine, Charité, Universitätsmedizin Berlin, Berlin, Germany

**Keywords:** Gender-specific, Gender-sensitive, Mental health services, Child psychiatry, Adolescent psychiatry

## Abstract

Gender differences in mental health emerge as early as in childhood and adolescence, highlighting the potential need for gender-specific child and adolescent mental health care. However, it is unclear how gender-specific child and adolescent mental health care is implemented and whether its’ approaches are useful. Therefore, this study reviews gender-specific interventions and their effectiveness for child and adolescent mental health. Five databases were searched for articles published between 2000 and 2021. In total, 43 studies were included. Most interventions were conducted in school (*n* = 15) or community settings (*n* = 8). Substance-related disorders (*n* = 13) and eating disorders (*n* = 12) were addressed most frequently. Most interventions targeted girls (*n* = 31). Various gender-specific aspects were considered, including gender-specific risk and protective factors (*n* = 35) and needs (*n* = 35). Although most interventions yielded significant improvements in mental health outcomes (*n* = 32), only few studies reported medium or large effect sizes (*n* = 13). Additionally, there was a lack of strong causal evidence derived from randomized controlled trials, calling for more rigorous trials in the research field. Nevertheless, our findings indicate that gender-specific mental health care can be a promising approach to meet gender-specific mental health needs.

**Supplementary Information:**

The online version contains supplementary material available at 10.1007/s10578-023-01506-z.

## Introduction

Gender, referring to the psychological, behavioral and sociocultural aspects associated with sex [1], is an important determinant for mental health outcomes and care. Several studies have revealed gender differences in terms of prevalence and clinical presentation for various mental health problems in children and adolescents such as eating disorders, anxiety disorders, conduct problems, and attention-deficit/hyperactivity disorder (ADHD) [2, 3]. For example, internalizing problems such as anxiety disorders occur more often among girls than boys [4], whereas externalizing problems such as conduct problems are more common in boys than in girls [5]. Furthermore, there are gender differences regarding coping strategies, symptomatology and medication [6–9]. There are also gender differences in child and adolescent mental health care regarding the frequency of service use [10], the age at referral [11], as well as in the willingness to use mental health care [12]: Compared to girls, boys are referred at a younger age [10, 11], whereas girls are more likely to obtain treatment in early adulthood [11]. Furthermore, adolescent boys are less willing to use mental health services than girls [12]. For various mental health problems, such as depressive symptoms [13, 14], substance use/abuse [15] or eating disorders [16], different risk and protective factors have been identified for girls and boys. Additionally, research on the etiology of mental health problems points towards the existence of gender-specific processes in their development [17, 18]. For example, rumination seems to contribute to the development of depression in girls, but not in boys [18].

In addition to this predominantly binary (girls vs. boys) and heteronormative view of gender differences, existing research demonstrates that transgender, gender variant or non-binary youth experience mental health problems, such as depression, suicidality and eating disorders, at an elevated rate compared to the general population [19–21]. Sexual and gender minorities (SGM), an umbrella term for individuals who do not identify as heterosexual or cisgender (e.g., transgender), face high levels of stigmatization and discrimination, and often adverse mental health outcomes [22].

Mental health problems emerging in these early life stages account for a large proportion of the global burden of disease [23], have economic long-term consequences [24], and often persist into adulthood [25, 26]. Additionally, research suggests that the persistence differs by gender, for instance, girls show a more persistent course of mood and anxiety disorders than boys [25]. In summary, there are several gender differences in mental health outcomes and care. Hence, considering gender aspects in child and adolescent mental health care is crucial for developing effective programs and thereby addressing gender-related mental health disparities early.

Programs that acknowledge gender roles and norms and consider and account for specific gender-related needs can be described as “gender-specific” [27, 28]. Furthermore, heteronormativity is an important gender-related aspect that should be addressed in gender-specific care [28], highlighting the need to consider not only aspects related to gender (e.g., female, transgender, etc.) but also to sexual identity (e.g., heterosexual, bisexual, etc.). Rationales for gender-specific interventions comprise (1) responding to gender-specific psychological aspects (e.g., gender-related needs and experiences), (2) providing a safe space and a supportive, empowering environment, (3) assuming a greater effectiveness of gender-specific compared to gender-non-specific interventions, and (4) thereby addressing gender disparities and contributing to the achievement of social justice [29]. Importantly, approaches that address one sex without addressing gender-specific aspects (mere single-sex interventions) are non-gender-specific programs [28].

Child and adolescent mental health care programs can also vary in the timing of intervention. A common distinction is made between prevention (before the onset of any disorder, focus on reducing risk factors and enhancing protective factors) and treatment programs (treating an already existing disorder) [30]. There are universal programs (targeting a whole population) and selective programs (targeting individuals or subgroups with a significantly higher risk of developing mental health problems) [31].

The development of gender-specific prevention programs for substance abuse began when substance abuse among girls increased in the mid-1990s, and studies showed that girls did not benefit from existing programs [15, 32]. A frequently addressed gender-specific aspect of these early programs was the focus on family relationships (a specific risk factor for girls) [15]. At this same time, the first gender-specific aggression interventions for girls were developed [32]. These treatment programs often focused on the different expression of aggression in girls vs. boys (relational vs. physical aggression) [32].

Despite these early developments, there are a few reviews of gender-specific child and adolescent mental health care for certain disorders (e.g., substance use/abuse [15]), settings (e.g., school [32]), and populations (e.g., SGM [33, 34]). However, a comprehensive review of gender-specific child and adolescent mental health care programs, without focusing on one specific mental health problem, setting, or population is lacking to better understand how their implementation and effectiveness might differ. Therefore, the purpose of this systematic review is to provide an overview of gender-specific child and adolescent mental health care by collating and critically appraising the existing research. It aims (1) to summarize and evaluate various gender-specific mental health care programs, (2) to identify the gender-specific aspects commonly addressed in these programs and (3) to synthesize their effectiveness, in order to derive implications for clinicians and health care providers. Further objectives are to identify gaps in knowledge and research, in order to make recommendations for future research.

## Methods

### Eligibility Criteria

**Fig. 1:**
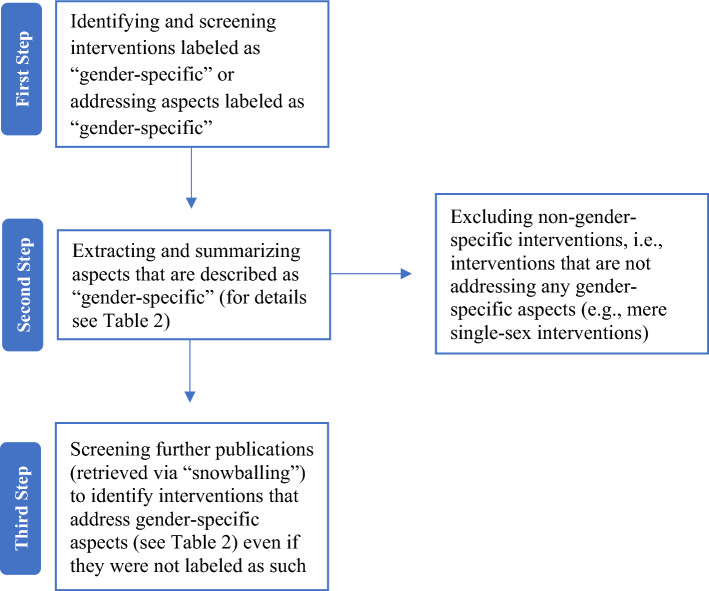
Process of identifying gender-specific interventions

To meet inclusion criteria, publications had to: (1) be published in peer-reviewed journals or books; (2) be written in English or German (due to limited language, time and financial resources); (3) be published between January 2000 and May 2021; (4) describe or evaluate interventions for children and adolescents (*M* ≤ 21 years, to include also late adolescence [35], and a minority of adults, to ensure the focus on children and adolescents and simultaneously acknowledge the fact that adolescent mental health care sometimes comprises young adults); (5) describe or evaluate a specific gender-specific intervention (any kind of intervention addressing gender-specific aspects; Fig. 1); and (6) address mental health care in different settings (e.g., child and adolescent psychiatric, psychological or psychotherapeutic care; school settings). Furthermore, articles were excluded if they: (1) were news, editorials, commentaries, reviews or case reports; (2) described or evaluated a non-gender-specific intervention (Fig. 1); (3) described or evaluated a gender-affirming intervention; or (4) did not focus on mental health.

### Search Strategy

The review adheres to the Preferred Reporting for Systematic Reviews and Meta-Analyses (PRISMA) guidelines [36]. We conducted a systematic search in PubMed, Social Science Citation Index, PsycInfo, PSYNDEX and Cochrane Library. Databases were searched by combining relevant text words and medical subject headings. We adapted the search strategies to each database (Appendix A). The search strategies covered the following topics: gender specificity, mental health care, and children and adolescents.

Electronic searches were complemented by reference list screening, citation tracking and hand searches in Google Scholar. Additionally, reviews with various focuses were screened [15, 16, 32–34, 37–46] to find gender-specific interventions which were not specifically described as “gender-specific” or with related terms and therefore not retrieved via electronic database search.

#### Study Selection

After removing the duplicates (with EndNote and manual checking), we transferred the records to Rayyan [47], an online software for the screening process. We identified potentially relevant articles by reviewing the titles, abstracts and keywords of all retrieved records. Three reviewers (LG, LH, MG) performed the title-abstract screening, wherein one reviewer (LH) screened all titles and abstracts and two reviewers (LG, MG) each additionally screened 10% with substantial agreement ($\kappa$ = 0.62–0.73) [48]. The full-texts were screened by three reviewers: One reviewer (LH) assessed each full-text and two reviewers (LG, MG) each additionally assessed 13% with moderate to substantial agreement ($\kappa$ = 0.48–0.72) [48]. Discrepancies between the raters were discussed until consensus was reached.

### Data Extraction

One reviewer (LH) extracted data from the included studies using a data extraction table with the following headings: author(s) and year of publication, country, setting, intervention type, description and components, gender specificity, target group, participants, age, study design, main outcome measures, main outcomes, and rating of study quality. Two reviewers cross-checked the data extraction (LG, MG).

### Critical Appraisal

The included studies were critically appraised using the Quality Assessment Tool for Quantitative Studies (QATQS) [49]. In previous evaluations the QATQS was found to be valid and reliable [50]. The QATQS rates methodologic study quality based on six criteria: selection bias, study design, confounders, blinding, data collection method, and withdrawals and drop-outs. Each study receives a global rating as weak, moderate, or strong. Three authors independently evaluated the studies. Discrepancies were discussed and resolved through consensus.

### Synthesis of Effectiveness

Effect sizes were extracted and transformed to Cohen’s *d*. Several studies reported data on different mental health outcomes. In these cases, the effect sizes were averaged within each study. Effect sizes were transformed with the Psychometrica online calculator [51]. Studies that reported non-significant results (without giving additional information) were set to an effect size of *d* = 0.00, a procedure which yields conservative effect sizes [52]. If effect sizes were reported as small, medium or large without specifying which effect sizes were computed, we applied the lower limits of *d* according to Cohen’s conventions [53].

## Results

**Fig. 2:**
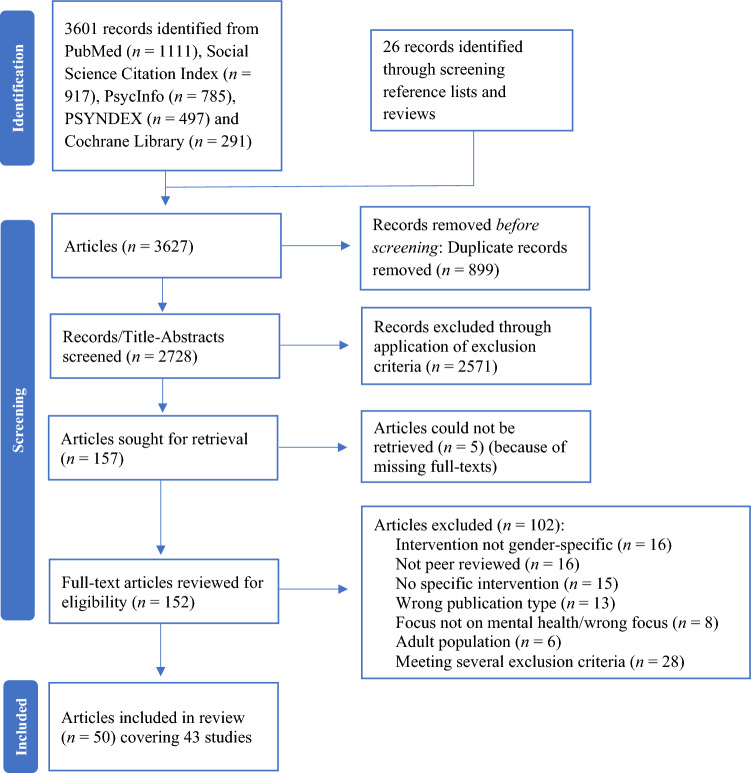
Flow diagram of literature searches and review process results

Searches yielded 3,627 records, of which 157 articles were potentially relevant for inclusion in this review (Fig. 2). After full-text screening, 50 publications were included, covering a total of 43 studies (Appendix B).

### Study Characteristics

The included 43 articles were published between 2001 and 2020 (Table 1). Almost all studies (*n* = 41) were conducted in North America or Europe. The interventions were mostly carried out in school (*n* = 15) or community settings (*n* = 8). Most of the studies assessed either universal prevention programs (*n* = 18) or treatments (*n* = 16). The most commonly targeted mental health problems were related to the DSM-5 [54] diagnostic categories “Substance-Related and Addictive Disorders” (*n* = 13) and “Feeding and Eating Disorders” (*n* = 12). Target groups were predominantly girls (*n* = 31) and adolescents (age ≥ 12, *n* = 35). In total, 19 studies were clinical controlled trials (i.e., experimental clinical studies without randomization), 12 cohort studies (i.e., one-group pretest–posttest), six randomized controlled trials (RCTs), four cohort analytic studies (i.e., two-group pretest–posttest), one interrupted time series, and one mixed-methods study. The study designs were classified according to the QATQS dictionary [55]. Sample sizes ranged from 7 to 2516 participants (*M* = 380).

**Table 1:** Study characteristics of the 43 included studies

Characteristics	All studies (*N* = 43)	
	*n*	Valid %
Publication date (*N* = 50)		
2000–2005	11	22.0
2006–2010	14	28.0
2011–2015	15	30.0
2016–2020	10	20.0
Location		
North America	30	69.8
Europe	11	25.6
Africa	1	2.3
Zealandia/New Zealand	1	2.3
Setting		
School	15	34.9
Community	8	18.6
Individual	6	14.0
Family	5	11.6
Residential facility	5	11.6
Clinical	2	4.7
Juvenile justice system	2	4.7
Type		
Universal prevention	18	41.9
Treatment	16	37.2
Universal intervention	4	9.3
Selective prevention	3	7.0
Adapting mental health care (system-level)	2	4.7
Targeted mental health problem		
Substance-related and addictive disorders	13	30.2
Feeding and eating disorders	12	27.9
Disruptive, impulse-control, and conduct disorders	5	11.6
Trauma- and stressor-related disorders	5	11.6
Mood disorders	3	7.0
Mental health care setting	2	4.7
Other mental health problems	2	4.7
Personality disorders	1	2.3
Target population/participants		
All girls	31	72.1
Mixed-gender groups (e.g., girls and boys)	6	14.0
All SGM^a^	4	9.3
All boys	2	4.7
Age of the target population/participants^b^		
Children (approx. 5–11 years)	13	–
Adolescents (approx. 12–21 years)	35	–
Young adults (approx. 22–25 years)	3	–
Adults (approx. 26–55 years)^c^	1	––
Study design		
Clinical controlled trial	19	44.2
Cohort (one-group pretest–posttest)	12	27.9
Randomized controlled trial	6	14.0
Cohort analytic (two-groups pretest–posttest)	4	9.3
Interrupted time series	1	2.3
Mixed methods	1	2.3
Sample size		
1–100	20	46.5
101–500	13	30.2
501–1000	6	14.0
1001–2000	2	4.7
2001–3000	2	4.7

The rows of each study characteristic are presented in descending order of frequency, except for the publication date and age

^a^Sexual and gender minority (SGM) youth were allocated to one group although we acknowledge that SGM youth are a heterogeneous group with diverse gender allocations, sexual identities and experiences

^b^Some of the included interventions targeted more than one age group

^c^There was one intervention targeting adult staff working with adolescent females

### Study Quality

For two studies the study quality could not be rated because several criteria of the QATQS were not applicable. The study quality among the remaining 41 studies was heterogeneous: 14 studies (34.1%) received a weak, 19 studies (46.3%) a moderate and eight studies (19.5%) a strong rating.

### Implementation of Gender-Specific Aspects

We grouped the studies depending on their targeted mental health problem as the interventions for each diagnostic category had similar gender-specific approaches. Study details and references of each intervention are presented in Appendix B.

#### Mood Disorders

In total, we identified three studies in the field of mood disorders [56–58], all studies (*n* = 3) targeted SGM youth of diverse sex and gender. One study each was conducted in a community [56], family [57] and individual setting [58]. All interventions were treatments for depression/suicidality [56–58].

Gender-specific aims of all treatments (*n* = 3) were to meet the mental health needs of depressed/suicidal SGM youth. Treatment programs attended to common issues and situations, such as invalidating reactions or bullying due to SGM identity [56–58]. Furthermore, most treatments (*n* = 2) addressed gender-specific risk and protective factors (e.g., family support) [56, 57], used gender-specific role models and facilitators (e.g., SGM community members) [56, 58], and provided future support and resources (e.g., allies) [56, 57].

One example is “Rainbow SPARX”, a computerized cognitive-behavioral therapy (CBT) for depression, adapted to the needs of SGM youth [58]. The gender-adapted version provides the option to create gender non-conforming avatars and addresses relevant situations (e.g., coming out) and challenges (e.g., harassment) for SGM individuals [58].

#### Trauma- and Stressor-Related Disorders

Five studies reported interventions for trauma- and stressor-related disorders [59–63]. The target populations were mainly girls (*n* = 4) [59–62]. Most interventions (*n* = 4) were community-based [60–63] and all interventions were treatments for sexually abused or traumatized youth [59–63].

All treatments (*n* = 5) aimed to meet gender-specific mental health needs of sexually abused or traumatized youth [59–63], for example by considering the heightened risk for a history of sexual abuse among incarcerated girls [59]. Additionally, most treatments (*n* = 4) addressed common risk (e.g., guilt and shame) and protective factors (e.g., skills and individual strengths) of these youth [59–61, 63].

One example is “Project Kealahou”, providing a gender- and culturally-responsive system of care to meet the mental health needs of traumatized girls [62]. Girls can participate in prosocial activities and girls groups. The treatment, which is mainly delivered by female staff, aims to provide a safe space for this vulnerable population [62].

#### Feeding and Eating Disorders

We found 12 studies for eating disorders [64–79], of which most were for girls (*n* = 10) [65–75, 78, 79] and delivered in school settings (*n* = 10) [64–71, 76–79]. Two interventions addressed mixed-gender groups (girls and boys) [64, 76, 77]. Four studies evaluated universal interventions [64, 66, 68, 69] and eight prevention programs [65, 67, 70–79].

First, all universal interventions (*n* = 4) targeted the body image by reflecting on female beauty ideals and myths, which are considered risk factors for the development of eating disorders [64, 66, 68, 69]. Additionally, most universal interventions (*n* = 3) taught body acceptance [64, 68, 69]. One example is “Happy Being Me”, a gender-adapted intervention for both girls and boys. The intervention reflects both female and male beauty ideals and promotes body acceptance [64].

Second, prevention programs (*n* = 8) focused on female beauty ideals and the sociocultural pressure to be thin as gender-specific risk factors [65, 67, 70–79]. One example is the universal prevention program “PriMa” that addresses several risk and protective factors for anorexia nervosa in girls, such as female beauty ideals, media literacy and body images [78, 79]. The program targets only adolescent girls, given the need for effective prevention programs in this high-risk population [78, 79].

#### Disruptive, Impulse-Control, and Conduct Disorders

Five studies evaluated interventions for aggression or conduct problems [80–84]. All interventions were treatments for girls and were delivered in residential facilities (*n* = 3) [80–82] or community settings (*n* = 2) [83, 84].

The gender-specific focus of all treatments (*n* = 5) was to attend to gender-specific issues since aggression is often expressed differently in girls compared to boys [80–84], with relational aggression being more pronounced among girls [80, 82]. Additionally, most studies (*n* = 4) evaluated programs that were explicitly developed or gender-adapted due to the lack of treatments for aggression in adolescent girls [80–82, 84].

One example is the intervention “A Girls’ Relationship Group” [80, 82]. The treatment takes risk and protective factors (e.g., experiences of violence and coping strategies), relational aspects of aggression, gender-specific conflicts and situations, and gender-role socialization into account. Furthermore, participating girls are empowered and positive self-images are promoted [80, 82].

#### Substance-Related and Addictive Disorders

In total, 13 studies investigated interventions for substance use/abuse [85–100]. Most interventions (*n* = 10) targeted girls [85–87, 90–96, 98–100]. The settings were heterogeneous, with most studies conducted in school (*n* = 4) [85, 86, 88, 89, 92], family (*n* = 4) [87, 93–95] or individual (*n* = 3) [96–99] settings. The studies comprised 12 prevention [85–89, 91–100] and one treatment study [90, 91].

First, most prevention programs (*n* = 11) addressed gender-specific risk and protective factors for substance abuse, such as stress or the mother-daughter relationship [85–89, 91, 93–100]. Furthermore, most studies aimed to meet gender-specific needs (*n* = 10) [85–89, 91, 94–96, 98–100]. One example is the internet-delivered prevention program for Asian American girls that addresses specific risk and protective factors (e.g., depressive moods and self-efficacy) to meet the mental health needs of this underserved minority group [87]. Additionally, the program aims to strengthen the mother-daughter relationship and addresses other common issues (e.g., body image) [87].

Second, we identified one treatment program named “HEART” which is a residential substance abuse treatment for girls [90, 91]. It focuses on the multifaceted needs, issues, and risk factors (e.g., other mental health problems, peer influence, abuse history) of these girls, considers sociocultural influences, feminist perspectives and aims to empower the participants [90, 91].

#### Personality Disorders

We found one study describing a treatment for personality disorders: “ACTiv” is a gender-adapted version of the dialectical behavior therapy for adolescents (DBT-A) [101]. It is tailored to the needs and issues of adolescent boys with borderline symptoms by considering the elevated aggressiveness and impulsiveness in boys compared to girls, and by using a more action-oriented approach than the original DBT-A [101].

#### Other Mental Health Problems

Two interventions [102, 103] targeted other mental health problems: Hampel et al. [102] evaluated a school-based universal stress prevention program for girls and boys. Gender-specific needs, coping strategies, role models, situations and gendered stereotypes are important aspects of the program [102]. The other intervention named “Progress” is a community-based treatment program for adolescent girls with at risk-behavior. The program addresses developmental tasks and issues of adolescent girls [103].

#### Mental Health Care Setting

We found two studies addressing gender-specific aspects in the mental health care setting [104, 105]. Crable et al. [104] evaluated a trauma-informed training curriculum for staff members working with adolescent girls in residential care. The goal of the training is to increase the staff’s knowledge and awareness of the girls’ mental health needs and risk and protective factors [104]. Guss et al. [105] developed and added gender-related questions to clinic intake forms that included the correct name, pronouns, gender identity and birth-assigned sex. The intervention aims to better identify transgender adolescents in order to support them properly and meet their mental health needs.

## Summary of Gender-Specific Aspects

In summary, gender-specific risk and protective factors (*n* = 35; e.g., female beauty ideals), mental health needs (*n* = 35; e.g., of sexually abused youth) as well as tasks, challenges and issues (*n* = 27; e.g., developmental tasks of adolescent girls) were the most commonly addressed gender-specific aspects in the included studies (*n* = 43; see Table 2 for more details on gender-specific aspects).

**Table 2:** Gender-specific aspects addressed in included studies (*N* = 43), classified by diagnostic category according to DSM-5

	MD (*n* = 3)	TSRD (*n* = 5)	FED (*n* = 12)	DICD (*n* = 5)	SRAD (*n* = 13)	PD (*n* = 1)	OMHP (*n* = 2)	MHCS (*n* = 2)	Total (*N* = 43)
	*n* Studies	*n* Studies	*n* Studies	*n* Studies	*n* Studies	*n* Studies	*n* Studies	*n* Studies	*n* (%)
Gender-specific aspects									
Addressing gender-specific risk and protective factors (e.g., stress and mother-daughter-relationship for SRAD in girls)	2 [56, 57]	4 [59–61, 63]	12 [64–79]	4 [80, 82–84]	12 [85–91, 93–100]	–	–	1 [104]	**35** (81.4%)
Meeting gender-specific (mental health) needs (e.g., developing effective programs to prevent FED in high-risk girls)	3 [56–58]	5 [59–63]	8 [66–70, 72–75, 78, 79]	4 [80–82, 84]	11 [85–92, 94–96, 98–100]	1 [101]	1 [102]	2 [104, 105]	**35** (81.4%)
Attending to gender-specific tasks, challenges, and issues (e.g., developmental tasks such as the onset of puberty)	3 [56–58]	2 [59, 61]	2 [65, 71]	5 [80–84]	11 [85–87, 89–92, 94–96, 98–100]	1 [101]	2 [102, 103]	1 [105]	**27** (62.8%)
Reflecting on gender roles and norms & gendered stereotypes (e.g., female beauty ideals and body norms)	1 [58]	–	12 [64–79]	2 [80, 82]	3 [85, 86, 89–91]	–	1 [102]	–	19 (44.2%)
Implementing gender-specific role models and facilitators (e.g., female peer role models as characters in interventions for girls)	2 [56, 58]	2 [60, 62]	2 [72, 73]	–	7 [90–96, 98, 99]	–	1 [102]	–	14 (32.6%)
Empowerment, body positivity/acceptance and affirming gender (e.g., promoting body acceptance in FED interventions)	1 [56]	2 [59, 63]	6 [64, 65, 68, 69, 71, 78, 79]	2 [80, 83]	1 [90, 91]	–	–	1 [104]	13 (30.2%)
Incorporating gender-specific situations into intervention (e.g., outing for SGM youth)	3 [56–58]	2 [59, 61]	1 [76, 77]	3 [80–82]	–	–	1 [102]	–	10 (23.3%)
Creating safe spaces and relationships (e.g., female staff and girls groups)	1 [57]	3 [60, 62, 63]	–	1 [80]	1 [90, 91]	–	–	–	6 (14.0%)
Feminist or minority stress theories as theoretical frameworks (e.g., understanding the impact of socialization on girls’ aggression)	1 [56]	–	–	3 [80, 82, 83]	2 [90, 91, 97]	–	–	–	6 (14.0%)
Providing future gender-specific support & resources (e.g., allies or community support)	2 [56, 57]	–	–	–	–	–	–	–	2 (4.7%)

The rows are presented in descending order of frequency

*DICD* disruptive, impulse-control, and conduct disorders, *DSM-5* Diagnostic and Statistical Manual of Mental Disorders, 5th edition, *FED* feeding and eating disorders, *MD* mood disorders, *MHCS* mental health care setting, *OMHP* other mental health problems, *PD* personality disorders, *SGM* sexual and gender minority, *SRAD* substance-related and addictive disorders, *TSRD* trauma- and stressor-related disorders

### Effectiveness of Gender-Specific Interventions

For an overview of the effectiveness of gender-specific interventions, see Table 3. In total, 39 studies provided data on the effectiveness of the interventions regarding mental health outcomes. The applied methodology differed substantially. First, most studies that used control groups (*n* = 29) applied no-intervention control conditions (*n* = 17), and one single intervention a gender-neutral program as control condition [92]. Second, the duration between baseline and follow-up ranged from post-treatment to 8-year follow-ups. Lastly, the studies utilized various mental health measures.

**Table 3:** Effect sizes grouped by intervention type, targeted mental health problem and study design

Characteristics	All studies with reported effect sizes (*n* = 29)				
	No effect (*d* < 0.20)	Small effect sizes (*d* = 0.20–0.49)	Medium effect sizes (*d* = 0.50–0.79)	Large effect sizes (*d* ≥ 0.80)	Cohen’s *d*
	% (*n*) Studies	% (*n*) Studies	% (*n*) Studies	% (*n*) Studies	*M* (Range)
Intervention type					
Treatment	–	12.5% (1) [83]	25.0% (2) [81, 84]	62.5% (5) [56, 57, 60, 90, 91, 101]	1.10 (0.46–1.75)
Selective prevention	–	66.7% (2) [72–75]	–	33.3% (1) [100]	0.44 (0.23–0.80)
Universal intervention	50.0% (2) [68, 69]	25.0% (1) [66]	–	25.0% (1) [64]	0.39 (0.00–0.80)
Universal prevention	35.7% (5) [70, 71, 76–79, 102]	35.7% (5) [67, 85, 86, 93, 96, 97]	14.3% (2) [87, 89]	14.3% (2) [65, 98, 99]	0.31 (0.00–0.80)
Targeted mental health problem					
Trauma- and stressor-related disorders	–	–	–	100% (1) [60]	1.75 (–)
Personality disorders	–	–	–	100% (1) [101]	1.32 (–)
Mood disorders	–	–	–	100% (2) [56, 57]	1.26 (1.02–1.50)
Substance-related and addictive disorders	–	44.4% (4) [85, 86, 93, 96, 97]	22.2% (2) [87, 89]	33.3% (3) [90, 91, 98–100]	0.61 (0.20–1.42)
Disruptive, impulse-control, and conduct disorders	–	33.3% (1) [83]	66.6% (2) [81, 84]	–	0.58 (0.46–0.79)
Feeding and eating disorders	60.0% (6) [68–71, 76–79]	40.0% (4) [66, 67, 72–75]	–	16.7% (2) [64, 65]	0.26 (0.00–1.23)
Other mental health problems	100% (1) [102]	–	–	–	0.00 (–)
Setting					
School	53.8% (7) [68–71, 76–79, 102]	23.1% (3) [66, 67, 85, 86]	7.7% (1) [89]	15.4% (2) [64, 65]	0.27 (0.00–1.23)
Community	–	25.0% (1) [83]	25.0% (1) [84]	50.0% (2) [56, 60]	0.93 (0.46–1.75)
Individual	–	80.0% (4) [72–75, 96, 97]	–	20.0% (1) [98, 99]	0.37 (0.20–0.80)
Family	–	33.3% (1) [93]	33.3% (1) [87]	33.3% (1) [57]	0.84 (0.30–1.50)
Residential facility	–	–	50.0% (1) [81]	50.0% (1) [90, 91]	1.11 (0.79–1.42)
Clinical	–	–	–	100% (1) [101]	1.32 (–)
Juvenile justice system	–	–	–	100% (1) [100]	0.80 (–)
Study design					
Uncontrolled studies (e.g., cohort study)	14.3% (1) [102]	–	14.3% (1) [84]	71.4% (5) [56, 57, 60, 90, 91, 101]	1.07 (0.00–1.75)
Controlled studies (e.g., RCTs)	27.3% (6) [68–71, 76–79]	40.9% (9) [66, 67, 72–75, 83, 85, 86, 93, 96, 97]	13.6% (3) [81, 87, 89]	18.2% (4) [64, 65, 98–100]	0.37 (0.00–1.23)
Total	24.1% (7) [68–71, 76–79, 102]	31.0% (9) [66, 67, 72–75, 83, 85, 86, 93, 96, 97]	13.8% (4) [81, 84, 87, 89]	31.0% (9) [56, 57, 60, 64, 65, 90, 91, 98–101]	0.55 (0.00–1.75)

Only studies that reported effect sizes are listed. The rows within each category are ordered by size of the effect size measure (Cohen’s *d*)

*RCTs* randomized controlled trials

Most of the interventions (*n* = 32) yielded significant improvements in mental health outcomes at post-treatment or follow-up. However, only 29 studies reported effect sizes. The effects sizes were heterogeneous: Seven studies yielded no effects (24.1%) and nine small (31.0%), four medium (13.8%) and nine large (31.0%) effect sizes. The mean effect size was medium (*d* = 0.55). When looking only at controlled studies (e.g., RCTs, *n* = 22), six studies yielded no effects (27.3%) and nine small (40.9%), three medium (13.6%), and four large (18.2%) effect sizes. The mean effect size among controlled studies was small (*d* = 0.37).

Most studies that yielded no effects assessed school-based universal prevention programs targeting eating disorders (*n* = 4). Although most of the programs (with no effects) yielded improvements on the short-term (*n* = 4), the effects could not be maintained at follow-up.

Large effect sizes were especially found for interventions targeting mood, trauma- and stressor-related, and personality disorders (*n* = 4). Interventions with these targets also showed large mean effect sizes (*d* = 1.26–1.75), whereas interventions targeting feeding and eating disorders yielded on average small effect sizes (*d* = 0.26). Especially treatment programs yielded large effects (*n* = 5). Treatment programs also yielded larger mean effect sizes (*d* = 1.10) than universal interventions (*d* = 0.39) and selective and universal prevention programs (*d* = 0.44 and *d* = 0.31). With regard to the settings, large effect sizes were especially yielded in clinical settings and the juvenile justice system (*n* = 2, *d* = 0.80–1.32). Additionally, uncontrolled studies such as cohort studies yielded more often large effects (*n* = 5) and on average larger effect sizes than controlled studies such as RCTs (*d* = 1.07 vs. *d* = 0.37). One example for an intervention yielding large effects is the culturally modified trauma-focused CBT for posttraumatic stress symptoms in war-affected adolescent girls with a history of sexual abuse evaluated by O’Callaghan et al. [60]. At the 3-month follow-up, posttraumatic stress symptoms had significantly decreased, and psychosocial functioning improved (for more details, see Appendix B, page 2). Limitations of this RCT include a small sample size (*n* = 52) and the fact that the intervention group could not be compared to the waitlist-group at follow-up as the waitlist-group had already received treatment at this point in time [60].

## Discussion

This systematic review provides an overview of gender-specific child and adolescent mental health care. It aims at summarizing and evaluating gender-specific child and adolescent mental health care programs, identifying gender-specific aspects commonly addressed in these programs and synthesizing their effectiveness.

In total, we identified 43 studies. Most interventions were conducted in school or community settings which is in line with previous research showing that most youth with mental health problems are seen in non-healthcare settings [106, 107]. Furthermore, nearly half of the studies assessed prevention programs. We find it intriguing that gender-specific concepts play a role in the development of preventive interventions, but less so in treatment programs. Further light should be shed on whether this is a conceptual gap or whether actual gender-specific approaches are not labeled as such in the realm of treatment.

In our review, most interventions targeted substance-related and addictive disorders as well as eating disorders. Additionally, over 70% of the studies evaluated interventions exclusively for (mostly adolescent) girls. Taken together, these findings are not surprising since girls/women have traditionally been neglected in substance treatment which has resulted in the development of female-specific treatments in the last decades [15]. Regarding eating disorders, adolescent girls have been the focus of prevention programs for a long time because they are at particularly high risk [108]. This trend has contributed to the exclusion of male and SGM individuals from eating disorder prevention programs even though (particularly pre-adolescent) boys and SGM individuals have high risks, too [109]. Only two of 43 studies (one yielding a large effect, one with no effect) focused exclusively on boys, indicating a large research gap. Reasons for this research gap include the lack of awareness of male or non-female disordered eating and the emphasis in most research on anorexia nervosa, as binge eating disorders are more prevalent among males [109]. Furthermore, the focus on a small number of mental health problems provides important directions for future research.

Gender specificity was implemented differently depending on the targeted mental health problem and population. For instance, addressing family support and minority stressors seemed to be an important aspect in treating mood disorders among SGM youth [56, 58], whereas eating disorder interventions mainly targeted girls’ beauty ideals and body acceptance [64–79].

Most of the interventions addressed several gender-specific aspects, highlighting that gender-specific interventions should consider multiple factors and experiences that intersect with each other. For example, gender norms and ideals (e.g., “thin ideal”), developmental tasks (e.g., puberty and related body changes), and resulting gender-specific mental health needs (e.g., effective prevention of eating disorders) are interconnected. On the one hand, a specific gender involves not only experiences on biological, psychological, and sociocultural levels, but also individual experiences that differ in gender groups without being specifically related to gendered roles (e.g., the role of rumination in girls’ depression) [110]. On the other hand, addressing several gender-specific aspects at once may indicate a lack of an appropriate etiological basis, otherwise required by experts in the field [32].

When looking at both controlled and uncontrolled studies, as we did in the present review, our findings indicate that most gender-specific interventions led to an improvement in mental health outcomes (particularly in mood, trauma- and stressor-related, and personality disorders). However, only 29 of the 43 studies reported effect sizes and only few studies (*n* = 13) yielded medium or large effects. Additionally, the outcomes regarding the effectiveness were heterogeneous and a comparison between programs was challenging because they differed in modality, duration, and setting. The studies yielded on average medium effect sizes with treatment studies yielding larger effects than universal intervention and prevention programs. This is in accordance with other reviews reporting a “hierarchy in effect sizes” for different intervention types with treatment studies yielding larger effects than prevention studies [111]. Assuming that most participants in prevention programs or universal interventions do not have severe symptoms, a reduction of symptoms or significant effects may be harder to find in these populations. This may also partially explain why universal body image programs and eating disorder prevention programs often yielded small or no effects.

However, the results regarding the effectiveness must be interpreted cautiously due to methodological deficits for some studies including lacking control groups, small sample sizes, short-term follow-ups and poor study quality. For instance, when examining only controlled studies, the average effect size was small (*d* = 0.37), and 27% yielded no effects. Thus, by including uncontrolled studies (for the purpose of a comprehensive review of gender-specific programs in this field), the reported improvements may be attributed to other factors. In summary, strong causal evidence (derived from RCTs) is lacking, calling for more RCTs in this research field.

Two other limitations concern our search strategy. We did not include terms related to sex and gender (e.g., “boy”/“girl” or “transgender”) as adding these terms would have led to more than 20,000 additional results only in PubMed. With a more comprehensive search strategy, additional publications that do not include terms referring to “gender-specific” or “sex-specific” could have been detected. However, we were aware of this limitation and decided to use our search strategy primarily for two reasons: First, we wanted to know how gender-specific aspects are considered and thus also how “gender-specific” is currently understood in child and adolescent mental health care. Secondly, we conducted pilot searches to ensure that our search was specific, but not overly sensitive. These pilot searches revealed that adding sex- and gender-related terms produced a lot of noise and few relevant records. To counter this limitation, we also screened impactful reviews and reference lists. We found it intriguing that gender-specific interventions very often addressed mental health problems that are more prevalent in another gender group (e.g., female-specific interventions for substance disorders). We believe this could be symptomatic in areas in which regular interventions largely point to a specific gender group, maybe even with a gender-specific approach, but fail to label the interventions as gender-specific. These limitations might be addressed in future studies, for example by analyzing and comparing intervention manuals of single-gender and mixed-gender interventions. Additionally, we restricted our search to English and German publications due to limited language, financial and time resources. Consequently, publications in other languages were excluded and the samples of the included studies were mostly drawn from Western, Educated, Industrialized, Rich, and Democratic (WEIRD) countries—highlighting the dearth of studies from more diverse countries that impacts many different research fields [112].

Another limitation resulting from time constraints is that the screening process was primarily conducted by one author, while two other authors could only screen 10–13% each in the given time. However, the authors were in constant exchange and additionally consulted with the research group.

We identified only a single study comparing a gender-specific to a non-gender-specific intervention. Consequently, this review does not allow any conclusions regarding the comparative effectiveness of gender-specific vs. non-gender-specific interventions. On the one hand, this underlines an important research gap. On the other hand, it highlights that possible superior effectiveness is not the only rationale for gender-specific care. For instance, from a consumer perspective, a safe space where youth can be empowered and supported by peers and where they can share and explore their gendered experiences is also important [113].

Our review focused on gender in child and adolescent mental health care. Moreover, other social indicators, such as ethnicity and socioeconomic status, have an important impact on child and adolescent mental health outcomes and care [114, 115]. For this reason, we reported the ethnic/racial diversity for the included studies (Appendix B). Since these different social indicators interact with each other and shape individual experiences and health outcomes (intersectionality) [116], future research should assess how other (intersecting) social indicators are addressed in gender-specific care.

There are many approaches to advance the research on gender-specific care derived from this review. Researchers and mental health professionals can

1. evaluate the effectiveness of existing gender-specific interventions;
2. compare the effectiveness of non-gender-specific vs. gender-specific interventions (e.g., [92]);
3. adapt existing and effective interventions to gender-specific needs (e.g., [64, 76, 77]);
4. develop and evaluate gender-specific interventions for other mental health problems with known gender differences (e.g., ADHD, autism);
5. develop and evaluate gender-specific interventions for underserved populations (e.g., [58, 87]);
6. develop and evaluate gender-specific interventions that consider different social indicators (e.g., [60, 62, 87]);
7. both differentiate between gender and sex and assess sexual identities to address vulnerable SGM groups (e.g., [105]);
8. be encouraged to state to which degree an intervention that they report on is gender-specific; and
9. collect gender data and report gender-differentiated results in evaluation studies of interventions.

Further recommendations are directed at mental health professionals and providers who can:

1. teach their staff about gender-specific risk and protective factors and mental health needs (e.g., [104]); and
2. sensitize each other for their own gender-stereotyped behavior or thinking.

## Summary

In conclusion, this systematic review provides a comprehensive overview of the existing research on gender-specific child and adolescent mental health care. It summarizes and discusses various gender-specific programs that employed a wide range of methods. The study quality of many studies was limited and the effectiveness heterogeneous. Additionally, gender-specific interventions focused on few mental health problems. To further advance the field of gender-specific child and adolescent mental health care, researchers and mental health professionals are called upon to develop and evaluate gender-specific interventions targeting other mental health problems and populations, in order to foster gender equity in mental health.

## Supplementary Information

Below is the link to the electronic supplementary material.

Supplementary file1 (DOCX 25 kb)

Supplementary file2 (DOCX 96 kb)

Supplementary file3 (DOCX 39 kb)

Supplementary file4 (DOCX 34 kb)
