# Supplementary material for: Systematic Review of Gender-Specific Child and Adolescent Mental Health Care
Source: Child Psychiatry Hum Dev. 2023 Feb 27;55(6):1487–501. doi: 10.1007/s10578-023-01506-z (PMC11485121; doi:10.1007/s10578-023-01506-z)
Supplement: Supplementary file 1 — Supplementary file1 (DOCX 25 kb) [file 10578_2023_1506_MOESM1_ESM.docx]

**Appendix A:** Search strategies in different databases

Note: A comprehensive literature search in the following databases was run on 27 April 2021.

|  | **Social Science Citation Index (via Web of Science)** |
| --- | --- |
| #1 | TS=(child* OR adolesc* OR youth* OR p$ediatric* OR teen* OR juvenil*) OR KP=(children OR adolescents) |
| #2 | TS=(gender-sensi* OR gender-specific* OR sex-specific* OR gender-responsive* OR gender-related OR gender-targeted OR gender-aware* OR gender-informed OR gender-conscious* OR gender-orient* OR “gender sensi*” OR “gender specific*” OR “sex specific*” OR “gender responsive*” OR “gender related*” OR “gender targeted*” OR “gender aware*” OR “gender informed” OR “gender conscious*” OR “gender orient*” OR gendersensi* OR genderspecific* OR sexspecific* OR genderresponsive* OR genderrelated OR gendertargeted OR genderaware* OR genderinformed OR genderconscious* OR genderorient*) |
| #3 | TS=((("mental health" OR “mental healthcare” OR “mental wellbeing” OR “mental well-being” OR “behavio$ral health” OR “mental disorder*” OR “mental illness*” OR “mental problem*” OR “mental disease*” OR “mental health difficult*” OR psych*) AND (care OR “mental healthcare” OR counsel$ing OR service* OR program* OR intervention* OR treatment* OR therap* OR approach OR prevention* OR promot* OR p$ediatrics)) OR psychotherap* OR psychiatr* OR psychoeducation* OR Skills-training OR skillstraining or “skill* training”) OR KP=(“mental health services”) OR KP=("psychotherapy") OR KP=(“child psychiatry”) OR KP=(“adolescent psychiatry”) |
| #4 | #1 AND #2 AND #3 |

**Indexes=SSCI Timespan=2000-2021**

|  | **PubMed** |
| --- | --- |
| #1 | "child*"[tiab] OR "child"[MeSH Terms] OR "adolesc*"[tiab] OR "adolescent"[MeSH Terms] OR "youth*"[tiab] OR "pediatric*"[tiab] OR "paediatric*"[tiab] OR "teen*"[tiab] OR "juvenil*"[tiab] |
| #2 | "gender sensi*"[tiab] OR "gender specific*"[tiab] OR "sex specific*"[tiab] OR "gender responsive*"[tiab] OR "gender related"[tiab] OR "gender targeted"[tiab] OR "gender aware*"[tiab] OR "gender informed"[tiab] OR "gender conscious*"[tiab] OR "gender orient*"[tiab] OR "gendersensi*"[tiab] OR "genderspecific*"[tiab] OR "sexspecific*"[tiab] OR "genderresponsive*"[tiab] OR "genderrelated"[tiab] OR "gendertargeted"[tiab] OR "genderaware*"[tiab] OR "genderinformed"[tiab] OR "genderconscious*"[tiab] OR "genderorient*"[tiab] OR "gender-sensi*"[tiab] OR "gender-specific*"[tiab] OR "sex-specific*"[tiab] OR "gender-responsive*"[tiab] OR "gender-related"[tiab] OR "gender-targeted"[tiab] OR "gender-aware*"[tiab] OR "gender-informed"[tiab] OR "gender-conscious*"[tiab] OR "gender-orient*"[tiab] |
| #3 | (("mental health"[tiab] OR “mental health"[MeSH Terms] OR “mental healthcare” [tiab] OR "mental wellbeing"[tiab] OR "mental well-being"[tiab] OR "behavioural health"[tiab] OR "behavioral health"[tiab] OR "mental disorder*"[tiab] OR "mental disorders"[MeSH Terms] OR "mental illness*"[tiab] OR "mental problem*"[tiab] OR "mental disease*"[tiab] OR "mental health difficult*"[tiab] OR "psych*"[tiab]) AND ("care"[tiab] OR “mental healthcare”[tiab] OR "counseling"[tiab] OR "counselling"[tiab] OR "service*"[tiab] OR "program*"[tiab] OR "intervention*"[tiab] OR "treatment*"[tiab] OR "therap*"[tiab] OR "approach"[tiab] OR "prevention*"[tiab] OR "promot*"[tiab] OR "pediatrics"[MeSH Terms])) OR “mental health services"[MeSH Terms] OR "psychotherap*"[tiab] OR "psychotherapy"[MeSH Terms] OR “psychiatr*”[tiab] OR “child psychiatry”[MeSH Terms] OR “adolescent psychiatry”[MeSH Terms] OR "psychoeducat*"[tiab] OR "skills-training"[tiab] OR "skillstraining"[tiab] |
| #4 | (english[Filter] OR german[Filter]) AND (2000:2021[pdat]) |
| #5 | humans[Filter] AND (allinfant[Filter] OR child[Filter] OR adolescent[Filter] OR preschoolchild[Filter]) |
|  | #1 AND #2 AND #3 AND #4 AND #5 |

|  | **PsycInfo (via EBSCOHost)** |
| --- | --- |
| S1 | (AB child* OR adolesc* OR youth* OR p#ediatric* OR teen* OR juvenil*) OR (MJ child OR adolescent) |
| S2 | AB gender-sensi* OR gender-specific* OR sex-specific* OR gender-responsive* OR gender-related OR gender-targeted OR gender-aware* OR gender-informed OR gender-conscious* OR gender-orient* OR “gender sensi*” OR “gender specific*” OR “sex specific*” OR “gender responsive*” OR “gender related*” OR “gender targeted*” OR “gender aware*” OR “gender informed” OR “gender conscious*” OR “gender orient*” OR gendersensi* OR genderspecific* OR sexspecific* OR genderresponsive* OR genderrelated OR gendertargeted OR genderaware* OR genderinformed OR genderconscious* OR genderorient* |
| S3 | (AB (("mental health" OR “mental healthcare” OR “mental wellbeing” OR “mental well-being” OR “behavio#ral health” OR “mental disorder*” OR “mental illness*” OR “mental problem*” OR “mental disease*” OR “mental health difficult*” OR psych*) AND (care OR “mental healthcare” OR counsel#ing OR service* OR program* OR intervention* OR treatment* OR therap* OR approach OR prevention* OR promot* OR p#ediatrics)) OR psychotherap* OR psychiatr* OR psychoeducation* OR Skills-training OR skillstraining or “skill* training”) OR (MJ “mental health services” OR "psychotherapy" OR “child psychiatry” OR “adolescent psychiatry”) |
| S4 | PO Human AND LA (English OR German) |
| S5 | S1 AND S2 AND S3 AND S4 |

**PSYNDEX (via Livio)**

((DB=PSYNDEX(((child* OR adolesc* OR youth* OR pediatric* OR paediatric* OR teen* OR juvenil* OR Kind* OR Jugend*) OR MESH=child OR MESH=adolescents) AND (“gender-sensi*” OR “gender-specific*” OR “sex-specific*” OR “gender-responsive*” OR “gender-related” OR “gender-targeted” OR “gender-aware*” OR “gender-informed” OR “gender-conscious*” OR “gender-orient*” OR “gender sensi*” OR “gender specific*” OR “sex specific*” OR “gender responsive*” OR “gender related*” OR “gender targeted*” OR “gender aware*” OR “gender informed” OR “gender conscious*” OR “gender orient*” OR “gendersensi*” OR “genderspecific*” OR “sexspecific*” OR “genderresponsive*” OR “genderrelated” OR “gendertargeted” OR “genderaware*” OR “genderinformed” OR “genderconscious*” OR “genderorient*” OR geschlechtersensib* OR geschlechterspezifi* OR geschlechtssensib* OR geschlechtsspezifi*) AND ((("mental health" OR “mental healthcare” OR “mental wellbeing” OR “mental well-being” OR “behavioral health” OR “behavioural health” OR “mental disorder*” OR “mental illness*” OR “mental problem*” OR “mental disease*” OR “mental health difficult*” OR psych*) AND (care OR “mental healthcare” OR counseling OR counselling OR service* OR program* OR intervention* OR treatment* OR therap* OR approach OR prevention* OR promot* OR pediatrics OR paediatrics OR Versorgung* OR Behandlung* OR Prävention* OR Pädiatr* OR Beratung* OR Ansatz* OR Ansätze)) OR psychotherap* OR psychiatr* OR psychoeducation* OR Psychoedukation* OR Skills-training OR skillstraining or “skill* training” OR MESH=“mental health services” OR MESH=psychotherapy OR MESH=“child psychiatry” OR MESH=“adolescent psychiatry”))) AND PY=2000:)

**Cochrane Library**

(child* OR adolesc* OR youth* OR pediatric* OR paediatric* OR teen* OR juvenil*):ti,ab,kw AND (gender-sensi* OR gender-specific* OR sex-specific* OR gender-responsive* OR gender-related OR gender-targeted OR gender-aware* OR gender-informed OR gender-conscious* OR gender-orient* OR “gender sensi*” OR “gender specific*” OR “sex specific*” OR “gender responsive*” OR “gender related*” OR “gender targeted*” OR “gender aware*” OR “gender informed” OR “gender conscious*” OR “gender orient*” OR gendersensi* OR genderspecific* OR sexspecific* OR genderresponsive* OR genderrelated OR gendertargeted OR genderaware* OR genderinformed OR genderconscious* OR genderorient*):ti,ab,kw AND ((("mental health" OR “mental healthcare” OR “mental wellbeing” OR “mental well-being” OR “behavioral health” OR “behavioural health” OR “mental disorder*” OR “mental illness*” OR “mental problem*” OR “mental disease*” OR “mental health difficult*” OR psych*) AND (care OR “mental healthcare” OR counseling OR counselling OR service* OR program* OR intervention* OR treatment* OR therap* OR approach OR prevention* OR promot* OR pediatrics OR paediatrics)) OR psychotherap* OR psychiatr* OR psychoeducation* OR Skills-training OR skillstraining or “skill* training”):ti,ab,kw

**Search Limits:** Between Jan 2000 and

(Search word variations = off)

https://www.cochranelibrary.com/advanced-search/search-manager
