# Supplementary material for: Systematic Review of Gender-Specific Child and Adolescent Mental Health Care
Source: Child Psychiatry Hum Dev. 2023 Feb 27;55(6):1487–501. doi: 10.1007/s10578-023-01506-z (PMC11485121; doi:10.1007/s10578-023-01506-z)
Supplement: Supplementary file 2 — Supplementary file2 (DOCX 96 kb) [file 10578_2023_1506_MOESM2_ESM.docx]

**Appendix B**: Study details and references of each intervention, classified and ordered by diagnostic category according to DSM-5

Mood disorders

| **Author(s) and year of publication** | **Country** | **Setting** | **Intervention type** | **Interventiondescription** | **Intervention components** | **Gender-specificity** | **Target group** | **Participants** | **Age**  (in years) | **Study design** | **Main outcome measures**  (selection) | **Main outcomes**  (selection) | **Rating of study quality** |
| --- | --- | --- | --- | --- | --- | --- | --- | --- | --- | --- | --- | --- | --- |
| Craig and Austin (2016) | Canada | Community | Treatment | Adapted CBT group for depression (“AFFIRM”)  delivered by trained SGM facilitators | 8 modules: CBT and psychoedu-cation with a focus on the impact of minority stress | Exploring and validating SGM identity, addressing gender-specific needs, acknowledging minority stressors and identifying coping strategies, connecting to SGM allies | SGM adolescents | *N* = 30 (SGM)  57% Female, 37% Queer, 20% Male, 20% Non-Binary, 7% Transgender, 3% Two-Spirit, 3% Other  53% White, 27% Asian, 23% Mixed, 20% Black, 7% Indigenous, 7% Latin American | Range:  15 – 18  *M* = 17.14 | Cohort | Depressive symptoms (BDI-II)  Stress appraisal (SAMA) | **3-month follow-up:** Significant decreases in depressive symptoms ($n$^2^ = .23 / *d* = 1.09) and stress appraisal (subscale threat appraisal, $n$^2^ = .18 / *d* = 0.94) across time, with a mean large effect size (*d* = 1.02) | Moderate |
| Diamond et al. (2012) | USA | Family | Treatment | Modified ABFT for suicidality and depression delivered by clinical psychologists (“ABFT-LGB”) | Approx. 12 sessions: individual therapy, family therapy, parent education | Addressing gender-specific mental health needs and issues, improv-ing acceptance and safety in parent-child relationship, promoting access to affirmative resources | LGB adolescents with suicidal ideation | *N* = 10 (80% girls, 20% boys)  50% African American, 20% White, 20% Multi-racial, 10% other | Range:  14 – 18  *M* = 15.10 | Cohort | Suicidality (SIQ-JR)  Depression (BDI-II) | **3-month follow-up:** Significant decreases in suicidality (*d* = 2.10) and depressive symptoms (*d* = 0.90) across time, with a mean large effect size (*d* = 1.50) | Weak |
| Lucassen et al. (2015) | New Zealand | Individual | Treatment | Adapted CBT for depression, computer-delivered  (“Rainbow SPARX”) | 7 sessions: skills training,  games, psychoedu-cation | Option to create gender non-conforming avatars, addressing relevant situations (e.g., outing), stereotypes and challenges | Sexual minority adolescents and young adults with depression | *N* = 21 (48% girls, 52% boys)  71% New Zealand European, 10% Māori, 5% Pacific, 14% Asian | Range:  13 – 19  *M* = 16.50 | Cohort | Depressive symptoms (CDRS-R, RADS-2, & MFQ) | **3-month follow-up:** Significant decrease in depressive symptoms maintained, *ES* n.r. for follow-up | Moderate |

Note: Age groups: adolescents 12 – 21 years, young adults 22 – 25 years. Abbreviations: ABFT = Attachment-Based Family Therapy; BDI-II = Beck Depression Inventory-II; CBT = Cognitive Behavioral Therapy; CDRS-R = Children’s Depression Rating Scale-Revised; ES = effect size(s); LGB = Lesbian, Gay and Bisexual; MFQ = Mood and Feelings Questionnaire; RADS-2 = Reynolds Adolescent Depression Scale, Second Edition; n.r. = not reported; SGM = Sexual and Gender Minority; SAMA = Stress Appraisal Measure for Adolescents; SIQ-JR = Suicidal Questionnaire-Junior; SPARX = Smart, Positive, Realistic, X-factor thoughts.

Trauma- and stressor-related disorders

| **Author(s) and year of publication** | **Country** | **Setting** | **Intervention type** | **Intervention description** | **Intervention components** | **Gender-specificity** | **Target group** | **Participants** | **Age**  (in years) | **Study design** | **Main outcome measures**  (selection) | **Main outcomes**  (selection) | **Rating of study quality** |
| --- | --- | --- | --- | --- | --- | --- | --- | --- | --- | --- | --- | --- | --- |
| Arnold et al. (2003) | USA | Juvenile justice system | Treatment | Gender-specific CBT delivered by counselor | Approx. 26 weeks of: individual and group therapy, structured journaling, making art, role playing | Addressing the needs and issues of sexually abused girls as well as gender-specific risk and protective factors, empowerment, considering gender-specific risky situations | Incarcerated adolescent girls with a history of sexual abuse | *N* = 100 (100% girls)  IG: *n* = 45  CG: *n* = 55 (TAU)  35% White, 55% “Non-white” | Range:  12 – 17  *M* (n.r.) | Cohort analytic | Psychosocial functioning (MAAS) | **Post-treatment:** Significant improvements in psychosocial functioning in IG across time, *ES* n.r. | Moderate |
| O’Callaghan et al. (2013) | DR Congo | Community | Treatment | Culturally modified TF-CBT for posttraumatic stress symptoms delivered by non-clinical facilitators | 15 sessions: group therapy, individual sessions and psychoedu-cation | Involving female facilitator on sensitive topics (risk reduction of sexual violence), addressing gender-specific risk and protective factors as well as needs, creating a safe space for war-affected girls | War-affected adolescent girls with a history of sexual abuse (witnessed or experienced) | *N* = 52 (100% girls)  IG: *n* = 24  CG: *n* = 28 (waitlist) | Range:  12 – 17  *M* = 16.02 | RCT | Posttraumatic stress symptoms (UCLA PTSD RI)  Psychological functioning (AYPA) | **3-month follow-up:** Significant decrease in posttraumatic stress symptoms (*d* = 2.04) and significant improvements in psychosocial functioning (*d* = 0.95 – 2.45) in IG across time, with a mean large effect size (*d* = 1.75)^1^ | Strong |
| Smith et al. (2012) | USA | Community | Treatment | TF-CBT-based foster care for co-occurring trauma and delinquency (“MTFC+T”) delivered by trained caregivers and therapists | 30 sessions: individual therapy; add. family therapy, skill building, training, supervision and groups for foster parents, case management | Addressing the specific needs and issues of delinquent girls, addressing gender-specific risk and protective factors, considering gender-specific risky situations | Justice-involved adolescent girls with a trauma history | *N* = 30 (100% girls)  IG: *n* = 13  CG: *n* = 17 (TAU)  72% European American, 4% African American, 7% Native American, 7% Latino, 11% Multiracial | Range:  12 – 17  *M* = 15.31 | CCT | Trauma-related mental health symptoms (self-constructed score containing scales of the BSI, CBCL, K-SADS-PL and TSCC) | **12-month follow-up:** Significant lower levels of trauma-related mental health symptoms in IG relative to CG, *ES* n.r. | Moderate |
| Suarez et al. (2014) | USA | Community | Treatment | Trauma-informed, cultural-sensitive system of care (“Project Kealahou”) | Case management, community support, group activities and evidence-based mental health treatment | Addressing mental health needs of traumatized girls, girls groups, involving girls in prosocial activities, staff mostly female, providing safe relationships/  space | Preadolescent and adolescent girls with a trauma history and a diagnosed mental health disorder | *N* = 144 (100% girls)  57% Native Hawaiian, 55% White, 38% Chinese, 36% Filipino, 20% Japanese, 16% Samoan, 14% Native American/Alaska Native, 12% Puerto Rican, 10% Other Pacific Islander, 9% African American, 7% Mexican, 6% Other Asian, 2% Other Hispanic | Range:  11 – 18  *M* = 15.40 | Cohort | Social and emotional strengths (BERS-2C/2Y)  Behavioral and emotional problems (CBCL)  Anxiety (RCMAS-2)  Depression (RADS-2) | **6-month follow-up:** Significant improvement in social and emotional strengths, behavioral and emotional problems and depression across time, *ES* n.r. | Weak |
| Whaling et al. (2020) | USA | Community | Treatment | Multidisci-plinary trauma-focused program (“RISE”) delivered by trained staff | Survivor therapy (e.g., TF-CBT), yoga, making art, individual, group and family therapy, case manage-ment | Identifying individual risk and protective factors, addressing mental health and safety needs of population, empowerment | Female and SGM children, adolescents and young adults with a history of commercial sexual exploitation | *N* = 90  no information regarding gender or ethnic/racial diversity | Range: 10 – 24  *M* = 16.00 | Cohort | No mental health or psychosocial outcomes reported | n.r. | Not applica-ble^2^ |

Note: Age groups: children/ preadolescents 5 – 11 years, adolescents 12 – 21 years. Abbreviations: AYPA = African Youth Psychosocial Assessment; BERS-2C/2Y = Behavioral and Emotional Rating Scale, 2nd Edition; BSI = Brief Symptom Inventory; CBCL = Child Behavior Checklist; CBT = Cognitive Behavioral Therapy; CCT = Controlled Clinical Trial; CG = Control Group; DR = Democratic Republic; ES = effect size(s); IG = Intervention Group; K-SADS-PL = Schedule for Affective Disorders and Schizophrenia for School-Aged Children – Present and Lifetime Version; MAAS = Multidimensional Adolescent Assessment Scale; MTFC+T = Multidimensional Treatment Foster Care Plus Trauma; n.r. = not reported; RADS-2 = Reynolds Adolescent Depression Scale, Second Edition; RCMAS-2 = Revised Children’s Manifest Anxiety Scale, Second Edition; RISE = Resiliency Interventions for Sexual Exploitation; SGM = Sexual and Gender Minority; TAU = Treatment As Usual; TF-CBT = Trauma-Focused Cognitive Behavioral Therapy; TSCC = Trauma Symptom Checklist for Children; UCLA PTSD RI = The University of California at Los Angeles Posttraumatic Stress Disorder Reaction Index.

^1^ The groups (IG vs. CG) were not compared at follow-up, as the control group had received their intervention before the follow-up study was completed. As a consequence, the study was categorized as “uncontrolled” for the data synthesis of the effectiveness.

^2^ The study quality could not be rated because several criteria of the checklist were not applicable.

Feeding and eating disorders

| **Author(s) and year of publication** | **Country** | **Setting** | **Intervention type** | **Intervention**  **description** | **Intervention components** | **Gender-specificity** | **Target group** | **Participants** | **Age**  (in years) | **Study design** | **Main outcome measures**  (selection) | **Main outcomes**  (selection) | **Rating of study quality** |
| --- | --- | --- | --- | --- | --- | --- | --- | --- | --- | --- | --- | --- | --- |
| Bird et al. (2013) | UK | School | Universal intervention | Universal body image  intervention (“Happy Being Me”) delivered by first author | 3 sessions:  interactive elements | Intervention gender-adapted: reflecting on female *and* male beauty ideals, promotion body acceptance | Preadolescent girls and boys | *N* = 88 (47.7% girls)  IG: *n* = 43  CG: *n* = 45 (no interv.)  100% Caucasian | Range:  10 – 11  *M* (n.r.) | CCT | Body Satisfaction (BSVAS)  Thin-ideal internalization (SATAQ-3, Internalization-General subscale)  Restrained eating (TFEQ)  Emotional eating (TFEQ) | **3-month follow-up:** Significant increase in body satisfaction (*d* = -1.23) for girls in IG across time, with a large effect size (*d* = 1.23) | Weak |
| Favaro et al. (2005) | Italy | School | Universal prevention | Universal eating disorder prevention delivered by  trained teachers | 6 sessions: psychoedu-cation and interactive elements | Reflecting on female beauty ideals and body changes during adolescence for girls, promoting body acceptance | Adolescent girls | *N* = 129 (100% girls)  IG: *n* = 38  CG: *n* = 91 (no interv.)  no information regarding ethnic/racial diversity | Range:  16 – 18  *M =* 17.00 | CCT | Eating disorder (SCID – section eating disorders)  Eating attitudes (EAT-40) | **1-year follow-up:** Significant lower rates of developing an eating disorder in IG relative to CG (5 % vs. 11%) and significant improvements in eating attitudes (bulimia subscale, *ES* = 0.55) in IG across time, with a large effect size (*ES* could not be transformed to *d* due to missing information, *d* = 0.80 was applied) | Strong |
| Halliwell and Diedrichs (2014) | UK | School | Universal intervention | Universal dissonance body image intervention (“Body project”) delivered by author or doctoral student | 4 sessions: activities, psychoedu-  cation, and media literacy | Addressing mental health need of adolescent girls, reflecting on female beauty ideals and myths | Adolescent girls | *N* = 104 (100% girls)  IG: *n* = 52  CG: *n* = 52 (waitlist)  70% White, 7% Black, 12% Asian, 11% Mixed | Range:  12 – 13  *M* = 12.07 | CCT | Thin-ideal internalization  (TIIS)  Body dissatisfaction (BPSS)  Dietary restraint (DRES) | **1-week post intervention:** Significant decreases in thin-ideal interna-lization ($d$ = 0.36) and body dissatis-faction ($d$ = 0.29) in IG across time, with a mean small effect size (*d* = 0.33) | Weak |
| López-Guimerà et al. (2011) | Spain | School | Universal prevention | Universal eating disorder prevention delivered by authors | 4 sessions: psycho-education about nutrition, female beauty ideals and media literacy and interactive elements | Addressing mental health need of adolescent girls, reflecting on female beauty ideals and myths | Adolescent girls | *N* = 263 (100% girls)  IG 1: *n* = 57 (full treatment)  IG 2: *n* = 78 (partial treatment without nutrition component)  CG: *n* = 128 (no interv.)  89.7% Spanish, 5.3% Latin-American, 2.3% North-African, 0.7% mixed, 0.7% other | Range (n.r.)  *M* = 13.41 | CCT | Eating attitudes (EAT)  Influences of Aesthetic Body Ideal (CIMEC) | **6-month follow-up:** Significant improvement in eating attitudes (subscale Diet and preoccupation with food*; ES* = 0.37) for IG 2 relative to CG across time, with a small effect size (*ES* could not be transformed to *d* due to missing information, *d* = 0.20 was applied) | Moderate |
| McVey and Davis (2002) | Canada | School | Universal intervention | Universal body image intervention (“Every BODY Is a Somebody”) delivered by author | 6 sessions: psychoedu-cation, media literacy and interactive elements | Addressing mental health needs of adolescent girls, reflecting on female beauty ideals, promoting positive body image | Preadolescent girls | *N* = 263 (100% girls)  IG: *n* = 150  CG: *n* = 113 (no interv.)  48.9% Caucasian, 20% Asian, 11.1% African Canadian, 8.5% South Asian, 2.6% Native Canadian | Range (n.r.)  Median: 10.88 | CCT | Body image satisfaction (SIQYA – Body image)  Eating attitudes (children’s version EAT) | **12-month follow-up:** No effects | Strong |
| McVey et al. (2004)  (Replication McVey and Davis, 2002) | Canada | School | Universal intervention | Universal body image intervention (“Every BODY Is a Somebody”) delivered by author | 6 sessions: psychoedu-cation, media literacy and interactive elements | Addressing mental health needs of adolescent girls, reflecting on female beauty ideals, promoting body acceptance | Preadolescent girls | *N* = 258 (100% girls)  IG: *n* = 182  CG: *n* = 76 (no interv.)  44% Caucasian, 20% South Asian, 13% Asian, 9% African Canadian, 3% Native Canadian, 11% ‘other’ | Range (n.r.)  *M* = 11.18 | CCT | Body image satisfaction (SIQYA – Body image)  Self-esteem (RSES)  Eating attitudes (children’s version EAT) | **12-month follow-up:** No effects | Moderate |
| Raich et al. (2008) | Spain | School | Universal prevention | Universal eating disorder prevention (multimedia format) | 2 sessions: psycho-education about nutrition, female beauty ideals and media literacy | Addressing mental health needs of adolescent girls, reflecting on female beauty ideals and myths | Adolescent girls | *N* = 323 (100% girls)  IG 1: *n* = 79 (full treatment)  IG 2: *n* = 94 (partial treatment without nutrition component)  CG: *n* = 150 (no interv.)  no information regarding ethnic diversity | Range (n.r.)  *M* = 13.11 | CCT | Eating attitudes (EAT)  Influences of Aesthetic Body Ideal (CIMEC) | **6-month follow-up:** No effects | Moderate |
| Stewart et al. (2001) | UK | School | Universal prevention | Universal eating disorder prevention delivered by psychologists and psychiatrists | 6 sessions: psychoedu-cation and interactive elements | Addressing challenges of adolescence for girls and sociocultural pressure to be thin, promoting positive body image | Adolescent girls | *N* = 845 (100% girls)  IG: *n* = 459  CG: *n* = 386 (no interv.)  no information regarding ethnic diversity | Range:  13 – 14  *M =* 13.40 | CCT | Eating disorder pathology (4^th^ version EDE)  Eating attitudes (EAT) | **6-month follow-up:** No effects | Strong |
| Stice et al. (2006, 2008) | USA | Individual | Selective prevention | Selective eating disorder prevention delivered by author and graduate students | 2-3 sessions: psychoedu-cation and interactive elements (depending on IG) | Addressing mental health needs of adolescent girls, reflecting on female beauty ideals | Adolescent girls with body dissatisfaction | *N* = 481 (100% girls)  IG 1: *n* = 115 (Disso-nance Intervention)  IG 2: *n* = 117 (Healthy Weight Intervention)  CG 1: *n* = 123 (Expressive Writing)  CG 2: *n* = 126 (no interv.)  58% Caucasian, 19% Hispanic, 10% Asian, 6% African, 7% other or mixed | Range:  14 – 19  *M* = 17.00 | RCT | Thin-ideal internalization (IBSS)  Body dissatisfaction (BPSS)  Dieting (DRES)  Bulimic symptoms (EDE)  Eating pathology (EDDI) | **3-year follow-up:** Lower risk for eating pathology in both IGs relative to CG 2 (*HR* = 2.27 – 2.75) and significant improvement in thin-ideal internalization in IG 2 relative to CG across time (*d* = 0.23), with a small effect size (*d* = 0.23) | Strong |
| Stice et al. (2009, 2011) | USA | Individual | Selective prevention | Selective eating disorder prevention delivered by author and graduate students | 4 sessions: psychoedu-cation and interactive elements | Addressing mental health needs of adolescent girls, reflecting on female beauty ideals | Adolescent girls with body image concerns | *N* = 306 (100% girls)  IG: *n* = 139  CG: *n* = 167 (brochure control condition)  81% Caucasian, 9% Hispanic, 2% Asian, 2% African American, 6% other or mixed | Range (n.r.)  *M* = 15.70 | RCT | Thin-ideal internalization (IBSS)  Body dissatisfaction (BPSS)  Dieting (DRES)  Eating pathology (EDDI) | **3-year follow-up:** Significant decreases in eating pathology (*d* = 0.30) in IG relative to CG across time, with a small effect size (*d* = 0.30) | Strong |
| Weigel et al. (2015); Gumz et al. (2017) | Germany | School | Universal prevention | Universal AN, BN and BED prevention delivered by members of prevention center or research team | 3 sessions: psychoedu-cation and interactive elements | Intervention gender-adapted: reflecting on female *and* male Western beauty ideals & gender-adapted materials (e.g., difficult situations & coping strategies) | Adolescent girls and boys | *N* = 2342 (55.6% girls)  IG: *n* = 724  CG: *n* = 728 (no interv.)  31.1% migration background | Range:  14 – 17  *M* = 14.60 | RCT | Eating disorder pathology (children’s version EDE-Q) | **6-month follow-up:**  No effects | Strong |
| Wick et al. (2011);  Adametz et al. (2017) | Germany | School | Universal prevention | Universal AN prevention (“PriMa”) delivered by trained teachers | 9 sessions: psychoedu-cation and interactive elements | Addressing mental health needs of adolescent girls, reflecting on female beauty ideals, promoting a positive body image | Preadolescent and adolescent girls | *N* = 1553 (100% girls)  IG: *n* = 450  CG: *n* = 437 (no interv.)  (only *n* = 100 girls at follow-up)  no information regarding ethnic/racial diversity | Range:  11 – 13  *M* = 12.00  (at baseline) | Cohort analytic | Body self-esteem (FBek)  Eating attitudes (EAT) | **7-to 8-year follow-up:** No effects  (but significant decrease in body self-esteem in *CG*, but not in IG) | Moderate |

Note: Internalized beauty ideals were addressed as gender-specific risk factors in all interventions. Age groups: children/ preadolescents 5 – 11 years, adolescents 12 – 21 years. Abbreviations: AN = Anorexia Nervosa; BEE = Binge Eating Disorder; BN = Bulimia Nervosa; BPSS = Body Parts Satisfaction Scale; BSVAS = Body Satisfaction Visual Analogue Scale; CCT = Controlled Clinical Trial; CG = Control Group; CIMEC = Questionnaire of Sociocultural Influences on the Aesthetic Body Shape Model; DRES = Dutch Restrained Eating Scale; EAT = Eating Attitudes Test; EDDI = Eating Disorder Diagnostic Interview; EDE(-Q) = Eating Disorder Examination (Questionnaire); ES = effect size(s); FBeK = Fragebogen zur Beurteilung des eigenen Körpers [Body Image Assessment Questionnaire]; IG = Intervention Group; n.r. = not reported; NUT-Q = Nutrition Questionnaire; PriMa = Primary Prevention of Anorexia Nervosa in Preadolescent Girls; RCT = Randomized Controlled Trial; RSES = Rosenberg Self-Esteem Scale; SATAQ-3 = Sociocultural Attitudes Towards Appearance Questionnaire-3; SCID = Structured Clinical Interview for DSM-IV Axis I Disorders; SIQYA = Self-Image Questionnaire for Young Adolescents; TFEQ = Three Factor Eating Questionnaire; TIIS = Thin Ideal Internalization Scale.

Disruptive, impulse-control, and conduct disorders

| **Author(s) and year of publication** | **Country** | **Setting** | **Intervention type** | **Intervention**  **description** | **Intervention components** | **Gender-specificity** | **Target group** | **Participants** | **Age**  (in years) | **Study design** | **Main outcome measures**  (selection) | **Main outcomes**  (selection) | **Rating of study quality** |
| --- | --- | --- | --- | --- | --- | --- | --- | --- | --- | --- | --- | --- | --- |
| Cummings et al. (2004) | Canada | Residential facility | Treatment | Treatment for aggressive behavior delivered by graduate counseling interns (“A Girls’ Relationship Group”) | 8 sessions: psychoeduc-ational group, skills training, role plays, and activities | Meeting mental health needs of aggressive girls, addres-sing risk and protective factors for girls’ aggression, considering other forms of aggression in girls (relational) and gender-role socialization, addressing gender-specific situations, feminist understanding and empower-ment | Adolescent girls with aggressive behavior | *N* = 8 (100% girls)  75% Caucasian, 25% Native | Range:  12 – 16  *M* = 14.70 | Cohort | Antisocial beliefs and values (BAS) | **Post-treatment:** Significant decrease in antisocial beliefs and values across time, *ES* n.r. | Weak |
| Goldstein et al. (2018) | USA | Residential facility | Treatment | Anger management and aggression reduction treatment  (“JJAM”) delivered by psychologists | 16 sessions: group-based treatment, psychoedu-cation, skills training, roles plays, and activities | Adapting the CPP to the needs and issues of girls involved in the juvenile justice system, addressing gender-specific situations | Adolescent girls in residential juvenile justice placements | *N* = 70 (100% girls)  IG: *n* = 35  CG: *n* = 35 (TAU)  63% Black or African American, 26% Hispanic, 23% more than one race, 11% White, 3% Asian | Range:  14 – 20  *M* = 17.45 | CCT | Anger and aggression (NAS-PI, AQ, PCS) | **Post-treatment:** Significant decreases in anger and aggression ($partial n$^2^ = .11 - .19 / *d* = 0.70 – 0.97) in IG relative to CG across time, with a mean medium effect size (*d* = 0.79) | Moderate |
| Hoffman et al. (2004) | Canada | Residential facility | Treatment | Treatment for aggressive behavior delivered by counseling interns (“A Girls’ Relationship Group”) | 8 sessions: group treatment, psychoedu-cation, role plays, and. skills training | Addressing risk and protective factors for girls’ aggression as well as gender-specific situations, considering other forms of aggression in girls (relational) and gender-role socialization, feminist understanding | Adolescent girls with aggressive behavior | *N* = 12 (100% girls)  83% Caucasian, 17% Native | Range  12 – 16  *M* = 14.70 | Cohort | Antisocial beliefs and values (BAS)  Aggression (RDAS) | **Post-treatment:** Significant decrease in antisocial beliefs and values across time, *ES* n.r. | Weak |
| Pepler et al. (2010) | Canada | Community | Treatment | Treatment for aggressive behavior (“SNAP GC”) delivered by clinicians | 12 sessions: group course for girls (‘SNAP Girls Club’) and parenting group (“SNAPP”) | Addressing risk and protective factors for girls’ aggression and their issues, empowerment, guided by feminist perspective | Preadolescent girls with behavioral problems | *N* = 80 (100% girls)  IG: *n* = 45  CG: *n* = 35 (waitlist)  42% Caucasian, 23% African Canadian, 3% Pacific Islander, 3% Latino, 3% Native Canadian, 26% other | Range:  5 – 11  *M* = 8.60 | CCT | Emotional and behavioral functioning (CBCL, TRF) | **Post-treatment**: Significant decreases in emotional and behavioral problems (*d* = 0.41 – 0.51) in IG relative to CG across time, with a mean small effect size (*d* = 0.46) | Strong |
| Walsh et al. (2002) | Canada | Community | Treatment | Treatment for conduct problems  (“EGC”) | 22 sessions: group course for girls (‘SNAP Girls Club’), Parenting group (‘SNAPP’) and mother-daughter-group (‘GGUH’) | Adapting program to risk and protective factors for girls’ aggression and their mental health needs, implementing other gender-specific interventions | Preadolescent girls with behavioral problems | *N* = 98 (100% girls)  no information regarding ethnic/racial diversity | Range:  5 – 11  *M* (n.r.) | Cohort | Emotional and behavioral problems (SCIS) | **12-month follow-up:** Significant decreases in emotional and behavioral problems (subscales externa-lizing behavior *d* = 0.49, and social relations *d* = 0.51) across time, with a mean medium effect size (*d* = 0.50) | Weak |

Note: Age groups: children/ preadolescents 5 – 11 years, adolescents 12 – 21 years. Abbreviations: AQ = Aggression Questionnaire; BAS = Beliefs and Attitudes Scale; CBCL = Child Behavior Checklist; CG = Control Group; CPP = Coping Power Program; EGC = Earlscourt Girls Connection; ES = effect size(s); GGUH = Girls Growing Up Healthy; IG = Intervention Group; JJAM = Juvenile Justice Anger Management; NAS-PI = Novaco Anger Scale and Provocation Inventory; n.r. = not reported; PCS = Peer Conflict Scale; RDAS = Relational and Direct Aggression Scale; SCIS = Standardized Client Information Systems; SNAP = Stop Now And Plan; SNAP GC = Stop Now And Plan Girls Connection; SNAPP = Stop Now And Plan Parenting; TRF = Teacher Report Form.

Substance-related and addictive disorders

| **Author(s) and year of publication** | **Country** | **Setting** | **Intervention type** | **Intervention description** | **Intervention components** | **Gender-specificity** | **Target group** | **Participants** | **Age**  (in years) | **Study design** | **Main Outcome measures**  (selection) | **Main outcomes** | **Rating of study quality** |
| --- | --- | --- | --- | --- | --- | --- | --- | --- | --- | --- | --- | --- | --- |
| Elliot et al. (2004, 2008) | USA | School | Universal prevention | Universal substance use and disordered eating prevention (“ATHENA”) delivered by trained peers | 8 sessions: interactive elements, psychoedu-cation and nutrition information | Addressing gender-specific needs, issues, correlates and risk factors for substance use and disordered eating behavior; reflecting on societal pressure to be thin | Adolescent female athletes | *N* = 928 (100% girls)  IG: *n* = 457  CG: *n* = 471 (TAU)  92% White | Range (n.r.)  *M* = 15.40  (at base-line) | RCT | Disordered eating behavior (single items)  Substance use (single items) | **1-to 3-year follow-up:** Significant decreases in substance use in IG relative to CG across time (*OR* = 0.26 – 0.61 / *d* = -0.74 –  -0.27), with a mean small effect size (*d* = 0.42) | Weak |
| Fang and Schinke (2013) | USA | Family | Universal prevention | Universal, substance use prevention, internet-delivered | 9 sessions: activities and psychoedu-cation for daughters and their mothers | Addressing specific risk and protective as well as mental health needs and common issues of Asian American girls; strengthening mother-daughter relationship | Asian American preadolescent and adolescent girls and their mothers | *N* = 108 (100% girls)  IG: *n* = 56  CG: *n* = 52 (no interv.)  100% Asian American, 19% born outside of the USA | Range:  10 – 14  *M* = 13.10 | RCT | Drug refusal skills (adapted from DRS)  Substance use intentions (adapted from CNUDS)  30-day substance use (adapted from ADAS) | **2-year follow-up:** Significant improvement in drug refusal skills ($n$^2^ = .05 / *d* = 0.46) and significant decreases in substance use intentions and 30-day substance use ($n$^2^ = .03 – .43 / *d* = 0.35 – 1.74) in IG relative to CG across time, with a mean medium effect size (*d* = 0.72) | Moderate |
| Goldberg et al. (2000) | USA | School | Universal prevention | Universal substance use prevention delivered coaches and trained peers (“ATLAS”) | 8 – 14 sessions: psychoedu-cation, interactive elements, nutrition information and training sessions | Addressing gender-specific needs, risk and protective factors for male athletes’ substance use (alcohol, other illicit drug use, anabolic steroids) | Adolescent male athletes | *N* = 2516 (100% boys)  IG: *n* = 1371  CG: *n* = 1145 (received pamphlet)  79% White, 7% Mixed Heritage, 5% African American, 4% Hispanic, 4% Asian, 1% Native American | Range (n.r.)  M = 15.42 | CCT | Substance use and intentions (single items) | **1-year follow-up:** Significant decreases in substance use and intentions in IG relative to CG across time, *ES* n.r. | Moderate |
| Hinz et al. (2007) | Germany | School | Universal prevention | Universal nicotine use prevention and health promotion (“Stark im Leben” [“Strong in Life”]) delivered by trained teachers and peers | 9 – 12 sessions: psychoedu-cation, activities, skills training | Addressing gender-specific needs, developmental tasks and risk factors, reflecting on traditional masculinity among boys and female beauty ideals among girls | Adolescent girls and boys | *N* = 660  IG: *n* = 320  CG: *n* = 340 (no interv.)  no information regarding gender or ethnic/racial diversity | Range:  12 – 16  *M* = 13.60 | Cohort analytic | Self-confident behavior (SSES)  Masculinity ideology (single items)  Body image (single items)  Smoking past 30 days (single items) | **6-month follow-up:** Significant increase in self-confident behavior ($n$^2^ = .10 / *d* = 0.67) among boys and girls in IG relative to CG across time and significant improvements in masculinity ideology ($n$^2^ = .03 / *d* = 0.35) among boys in IG relative to CG across time, with a mean medium effect size (*d* = 0.53) | Moderate |
| Roberts-Lewis et al. (2010) & Welch et al. (2009) | USA | Residential | Treatment | Residential substance abuse treatment program (“HEART”) delivered by trained staff | 5 stages of treatment with CBT, group therapy and meetings, psycho-pharmaco-therapy, 12 steps program, feminist groups, family treatment | Focusing on the needs, issues and risk factors of incarcerated girls, considering sociocultural influences, empowerment of girls, feminist perspective, incorporating female role models into program | Incarcerated adolescent girls with diagnosed substance abuse or dependency | *N* = 30 (100% girls)  no CG  63% White, 30% African American, 7% other | Range (n.r.)  *M* = 15.00 | Cohort | Functioning (POSIT) | **Post-**  **intervention:**  Significant improvements in functioning in most domains (e.g., mental health, peer relations; partial $n$^2^ = .13 – .53 / *d* = 0.77 – 2.12) across time, with a mean large effect size (*d* = 1.42) | Weak |
| Schinke and Schwinn (2005) | USA | School | Universal prevention | Universal substance abuse prevention delivered by CD-ROM (“Girls and Stress”) | 1 session: psychoedu-cation, teaching stress management skills | Addressing gender-specific issues (stress) and needs, using female peer role models as characters and female narrator | Adolescent girls | *N* = 91 (100% girls)  IG: *n* = 47  CG: *n* = 44 (non-gender-specific intervention) | Range (n.r.)  *M* (n.r.)  seventh-grade (approx. 12 – 13 years old) | CCT | Stress management (single items)  Substance use (single items adapted from YRBS and ADAS) | **Post-intervention:** Significant decrease in substance use plans in IG relative to CG across time, *ES* n.r. | Weak |
| Schinke et al. (2009a) | USA | Family | Universal prevention | Universal substance use prevention computer-delivered | 14 modules: activities and psychoedu-cation for daughters and their mothers, skills training | Addressing protective factors for girls’ underage drinking, using animated female characters, strengthening mother-daughter relationship | Preadolescent and adolescent girls | *N* = 202 (100% girls)  IG: *n* = 101  CG: *n* = 101 (waitlist)  68% White, 14% Latina, 10% Black, 1% Asian and 8% other | Range: 10 – 13  *M* = 12.20 | CCT | Alcohol refusal skills (LSTQ)  Alcohol use and intentions (ADAS) | **2-month follow-up:** Significant improvements in alcohol refusal skills$(n$^2^ = .02 / *d* = 0.29) and significant decreases in alcohol use ($n$^2^ = .02 – .03 / *d* = 0.29 – 0.35) in IG relative to CG across time, with a mean small effect size (*d* = 0.30) | Moderate |
| Schinke et al. (2009b) | USA | Family | Universal prevention | Universal substance use prevention computer-delivered | 9 sessions: activities and psychoedu-cation for daughters and their mothers, skills training | Addressing mental health need, risk and protective factors for girls’ substance use, and gender-specific issues, strengthening mother-daughter relationship | Preadolescent and adolescent girls | *N* = 591 (100% girls)  IG: *n* = 252  CG: *n* = 339 (no interv.)  49% Black, 26% White, 25% Latina | Range: 11 – 13  *M* = 12.70 | CCT | Substance use past 30 days (ADAS) | **1-year follow-up:** Significant decrease in substance use in IG relative to CG across time, *ES* n.r. | Moderate |
| Schinke et al. (2011) | USA | Family | Universal prevention | Universal substance abuse prevention computer-delivered | 10 sessions: activities and psychoedu-cation for daughters and their mothers, skills training | Addressing mental health needs, risk and protective factors for Black and Hispanic girls’ substance use, and gender-specific issues, strengthening mother-daughter relationship | Black and Hispanic preadolescent and adolescent girls | *N* = 546 (100% girls)  IG: *n* = 212  CG: *n* = 334 (no interv.)  65% Black, 34% Latina | Range: 10 – 13  *M* = 12.75 | CCT | Substance use and intentions (ADAS) | **Post-**  **intervention:** Significant decrease in substance use intentions in IG relative to CG across time, *ES* n.r. | Weak |
| Schwinn et al. (2010) | USA, Canada | Individual | Universal prevention | Universal substance abuse prevention computer-delivered  (“RealTeen”) | 12 sessions: skills training, activities, psychoedu-cation | Addressing gender-specific mental health needs, tasks/  issues and risk factors, using female character | Adolescent girls | *N* = 236 (100% girls)  IG: *n* = 118  CG: *n* = 118 (no interv.)  60% White, 17% Black, 7% Latina, 7% Asian, 9 % other | Range (n.r.)  *M* = 14.00 | CCT | Substance use past 30 days (single items adapted from YRBS and ADAS) | **6-month follow-up:** Significant decrease in substance use (*d* = 0.19 – 0.20) in IG relative to CG across time, with a mean small effect size (*d* = 0.20) | Weak |
| Schwinn et al. (2015) | USA | Individual | Universal prevention | Universal substance use prevention, web-based | 3 sessions: skills training, activities, psychoedu-cation | Addressing gender-specific risk and protective factors, intervention guided by minority stress theory | SGM adolescents | *N* = 236 (51% female, 33% male, 16% queer/  fluid/other gender)  IG: *n* = 119  CG: *n* = 117 (no interv.)  62% White, 13% Hispanic, 10% Black, 8% Asian, 8% Other | Range:  15 – 16  *M* = 16.08 | CCT | Drug refusal skills (no further information)  Substance use past 30 days (no further information) | **3-month follow-up:** Significant improvements in drug refusal skills (*d* = 0.32) and significant decrease in ‘other’ drug use (*d* = 0.34) in IG relative to CG, with a mean small effect size (*d* = 0.33) | Moderate |
| Schwinn et al. (2018, 2019) | USA | Individual | Universal prevention | Universal substance abuse prevention, web-based  (“RealTeen”) | 9 sessions: skills training, activities, psychoedu-cation | Using peer role model as narrator and addressing gender-specific mental health needs, tasks/issues and risk factors for girls’ sub-stance abuse | Adolescent girls | *N =* 788 (100% girls)  IG: *n* = 396  CG: *n* = 392 (no interv.)  63% White, 17% Black, 15% Latino, 4% Asian, 16% other | Range:  13 – 14  *M* = 13.68 | CCT | Drug use (single items adapted from YRBS) | **3-year follow-up:** Significant decrease in cigarette use (RR = .146 – .353) in IG relative to CG across time, with a mean large effect size (RR = 4.84; *ES* could not be transformed to *d* due to missing information, *d* = 0.80 was applied) | Moderate |
| Walker et al. (2019) | USA | Juvenile justice system | Selective prevention | Selective substance use and delinquency prevention delivered by trained staff (“GOAL”) | 20 sessions: cognitive behavioral group sessions, role plays, psycho- and parent education | Addressing gender-specific needs, issues and protective factors, guided by principle of girls’ develop-ment and empowerment | Court-involved adolescent girls | *N* = 87 (100% girls)  IG: *n* = 57  CG: *n* = 30 (TAU)  47% White, 26% mixed ethnicity, 8% Black, 7% Latina, 2% American Indian, 9% other | Range:  12 – 17  *M* = 15.15 | Cohort  analytic | Substance use and consequences (DISA)  Risky behavior (Risky Behaviors subscale of HSQ) | **6-month follow-up:** Significant decrease in risky behavior (*ES* = 0.84) in IG relative to CG across time, with a large effect (*ES* could not be transformed to *d* due to missing information, *d* = 0.80 was applied) | Moderate |

Note: Age groups: children/ preadolescents 5 – 11 years, adolescents 12 – 21 years. Abbreviations: ADAS = American Drug and Alcohol Survey; ATHENA = Athletes Targeting Healthy Exercise and Nutrition Alternatives; ATLAS = Adolescents Training and Learning to Avoid Steroids; CBT = Cognitive Behavioral Therapy; CCT = Controlled Clinical Trial; CG = Control Group; CNUDS = Commitment to Not Use Drug Scale; DISA = Drug Involvement Scale for Adolescents; DRS = Drug Refusal Skills; ES = effect size(s); GOAL = Girls Only Active Learning; HEART = Holistic Enrichment for At-Risk Teens; HSQ = High School Questionnaire; IG = Intervention Group; IRR = Incidence Rate Ratio; LSTQ = Life Skills Training Questionnaire; n.r. = not reported; POSIT = Problem Oriented Screening Instrument for Teenagers; SGM = Sexual and Gender Minority; SSES = State Self-Esteem Scale; YRBS = Youth Risk Behavior Survey.

Personality disorders

| **Author(s) and year of publication** | **Country** | **Setting** | **Intervention type** | **Intervention description** | **Intervention components** | **Gender-specificity** | **Target group** | **Participants** | **Age**  (in years) | **Study design** | **Main outcome measures**  (selection) | **Main outcomes**  (selection) | **Rating of study quality** |
| --- | --- | --- | --- | --- | --- | --- | --- | --- | --- | --- | --- | --- | --- |
| Heider et al. (2017) | Germany | Clinical | Treatment | Adapted DBT-A for Borderline personality disorder delivered by therapists (“ACTiv”) | Approx. 6 weeks: individual and group therapy, activities, skills group, family therapy, parent group | Adapting DBT-A to specific needs and issues of male adolescents | Male adolescents with borderline personality disorder | *N* = 7 (100% boys)  100% German nationality | Range:  13 – 16  *M* = 14.00 | Cohort | Psychological symptoms and distress (SCL-90-R)  Emotion regulation (FEEL-KJ)  Aggressive behavior (K-FAF)  Self-injurious behavior (OUSI) | **Post-treatment:** Significant decrease in aggressive behavior (*d* = 1.10 – 1.53) across time, with a mean large effect size (*d* = 1.32) | Moderate |

Note: Age group: adolescents 12 – 21 years. Abbreviations: ACTiv = Achtsamkeit Coolness Training aktiv [Mindfulness Coolness Training active]; DBT-A = Dialectical Behavioral Therapy for Adolescents; FEEL-KJ = Fragebogen zur Erhebung der Emotionsregulation bei Kindern und Jugendlichen [Questionnaire for Emotional Regulation in Children and Adolescents]; K-FAF = Kurzfragebogen zur Erfassung von Aggressivitätsfaktoren [Short Questionnaire for Aggressiveness Factors]; SCL-90-R = Symptom Checklist-90-Revised; OUSI = Ottawa/Ulm Selbstverletzungsinventar [Ottawa/Ulm Self-Injury Inventory].

Other mental health problems

| **Author(s) and year of publication** | **Country** | **Setting** | **Intervention type** | **Intervention description** | **Intervention components** | **Gender-specificity** | **Target group** | **Participants** | **Age**  (in years) | **Study design** | **Main outcome measures**  (selection) | **Main outcomes**  (selection) | **Rating of study quality** |
| --- | --- | --- | --- | --- | --- | --- | --- | --- | --- | --- | --- | --- | --- |
| Hampel et al. (2008) | Germany | School | Universal prevention | Universal stress prevention delivered by (graduate) students | 7 sessions: psychoedu-cation, skills training for stress manage-ment,  interactive elements | Adapting non-gender-specific intervention (“AST”) by addressing gender-specific needs, issues and coping strategies, using gender-specific role models and situations, reflecting on gendered stereotypes | Preadolescent girls and boys | *N* = 80 (44% girls, 56% boys)  no information regarding ethnic/racial diversity | Range (n.r.)  *M* = 11.10 | Cohort | Stress management (SVF-KJ) | **1-month follow-up:** No effects | Moderate |
| Turner and Werner-Wilson (2008) | USA | Community | Treatment | Day-treatment program (“Progress”) delivered by staff | 16 weeks: skill training, group therapy, activities | Addressing developmental tasks and issues of adolescent girls | Adolescent girls with at-risk behavior | *N* = 66 (100% girls)  85% Caucasian, 6% African American, 3% Asian, 3% Hispanic, 3% Other | n.r. | Cohort | No mental health or psychosocial outcomes reported | n.r. | Not applica-ble^1^ |

Note: Age groups: children/ preadolescents 5 – 11 years, adolescents 12 – 21 years. Abbreviations: AST = Anti-Stress-Training; n.r. = not reported; SVF-KJ = Stressverarbeitungsfragebogen für Kinder und Jugendliche.

^1^ The study quality could not be rated because several criteria of the checklist were not applicable.

Mental health care setting

| **Author(s) and year of publication** | **Country** | **Setting** | **Intervention type** | **Intervention description** | **Intervention components** | **Gender-specificity** | **Target group** | **Participants** | **Age**  (in years) | **Study design** | **Main outcome measures**  (selection) | **Main outcomes**  (selection) | **Rating of study quality** |
| --- | --- | --- | --- | --- | --- | --- | --- | --- | --- | --- | --- | --- | --- |
| Crable et al. (2013) | USA | Residential facility | Adapting Mental Health Care (System-Level) | Gender-specific and trauma-informed training curriculum for staff | 8 modules: psychoedu-cation on trauma and teaching tools for helping traumatized adolescents | Addressing the mental health needs (esp. trauma) and risk and protective factors of adolescent females in residential care, empowerment | Direct care staff members working with adolescent girls | *N* = 40  (staff members)  IG: *n* = 20  CG: *n* = 20 | Range:  22 – 55 (staff members) | Inter-rupted time series | Satisfaction with training (Satisfaction Survey)  Knowledge (Survey of Knowledge) | **Post-training:** Contrary to hypotheses significant decrease in satisfaction, *ES* n.r. | Weak |
| Guss et al. (2020) | USA | Clinical | Adapting Mental Health Care (System-Level) | Adapting clinic forms | Adding gender-related questions to clinic intake forms: correct name, pro-nouns, gender identity and birth-assigned sex | Inclusion of gender questions to meet specific needs and issues of transgender adolescents | Adolescents referred to primary care | **Interviews:**  *N* = 21 (52%, cisgender, 33% transgender, 14% non-binary or genderqueer)  **Intervention:**  *N* = 1442  IG: *n* = 827 (after form was implemented)  CG: *n* = 615 (before form was implemented) | Range: 15 – 25  **Interviews:**  *M* = 18.80  39% White, 24% Black, 10% Asian, 19% Multi-racial | Mixed methods (inter-views & two retro-spective chart reviews) | Acceptability of new questions on intake forms (structured interviews)  Gender-related documentation (EHR documentation) | **Interviews:** Gender-related questions acceptable for most participants (*n* = 20/21)  **Intervention:** Significant increase in gender documentation in EHR | Weak^1^ |

Note: Age groups: adolescents 12 – 21 years, adults 26 – 55 years. Abbreviations: CG = Control Group; HER = Electronic Health Record; ES = effect size(s); IG = Intervention Group, n.r. = not reported.

^1^ We acknowledge that the applied quality-assessment criteria are only partly adequate for judging the mixed-method design of Guss et al. (2020), nonetheless we decided to rate the study for reasons of completeness and comparability.

**References**

Adametz L, Richter F, Strauss B, Walther M, Wick K, Berger U (2017) Long-term effectiveness of a school-based primary prevention program for anorexia nervosa: A 7-to 8-year follow-up. Eat Behav 25:42-50

Arnold EM, Kirk RS, Roberts AC, Griffith DP, Meadows K, Julian J (2003) Treatment of incarcerated, sexually-abused adolescent females: An outcome study. J Child Sex Abus 12(1):123-139

Bird EL, Halliwell E, Diedrichs PC, Harcourt D (2013) Happy Being Me in the UK: A controlled evaluation of a school-based body image intervention with pre-adolescent children. Body Image 10(3):326-334

Crable AR, Underwood LA, Parks-Savage A, Maclin V (2013) An examination of a gender-specific and trauma-informed training curriculum: implications for providers. Int J Behav Consult Ther 7(4):30-37

Craig SL, Austin A (2016) The AFFIRM open pilot feasibility study: A brief affirmative cognitive behavioral coping skills group intervention for sexual and gender minority youth. Child Youth Serv Rev 64:136-144

Cummings AL, Hoffman S, Leschied AW (2004) A psychoeducational group for aggressive adolescent girls. J Spec Group Work 29(3):285-299

Diamond GM, Diamond GS, Levy S, Closs C, Ladipo T, Siqueland L (2012) Attachment-based family therapy for suicidal lesbian, gay, and bisexual adolescents: a treatment development study and open trial with preliminary findings. Psychotherapy (Chic) 49(1):62-71

Elliot DL, Goldberg L, Moe EL, Defrancesco CA, Durham MB, McGinnis W et al (2008) Long-term outcomes of the ATHENA (Athletes Targeting Healthy Exercise & Nutrition Alternatives) program for female high school athletes. J Alcohol Drug Educ 52(2):73-92

Elliot DL, Goldberg L, Moe EL, Defrancesco CA, Durham MB, Hix-Small H (2004) Preventing substance use and disordered eating: initial outcomes of the ATHENA (athletes targeting healthy exercise and nutrition alternatives) program. Arch Pediatr Adolesc Med 158(11):1043-1049

Fang L, Schinke SP (2013) Two-year outcomes of a randomized, family-based substance use prevention trial for Asian American adolescent girls. Psychol Addict Behav 27(3):788-798

Favaro A, Zanetti T, Huon G, Santonastaso P (2005) Engaging teachers in an eating disorder preventive intervention. Int J Eat Disord 38(1):73-77

Goldberg L, MacKinnon DP, Elliot DL, Moe EL, Clarke G, Cheong J (2000) The adolescents training and learning to avoid steroids program: preventing drug use and promoting health behaviors. Arch Pediatr Adolesc Med 154(4):332-338

Goldstein NE, Giallella CL, Haney-Caron E, Peterson L, Serico J, Kemp K et al (2018) Juvenile Justice Anger Management (JJAM) Treatment for Girls: Results of a randomized controlled trial. Psychol Serv 15(4):386-397

Gumz A, Weigel A, Daubmann A, Wegscheider K, Romer G, Löwe B (2017) Efficacy of a prevention program for eating disorders in schools: a cluster-randomized controlled trial. BMC Psychiatry 17(1):293

Guss CE, Eiduson R, Khan A, Dumont O, Forman SF, Gordon AR (2020) “It'd Be Great to Have the Options There”: A Mixed-Methods Study of Gender Identity Questions on Clinic Forms in a Primary Care Setting. J Adolesc Health 67(4):590-596

Halliwell E, Diedrichs PC (2014) Testing a dissonance body image intervention among young girls. Health Psychol 33(2):201-204

Hampel P, Jahr A, Backhaus O (2008) Geschlechtsspezifisches Anti-Stress-Training in der Schule. Prax Kinderpsychol Kinderpsychiatr 57(1):20-38

Heider J, Fleck A, Peteler C, Anker S, Lieb S, Behrens M et al (2017) Dialektisch-Behaviorale Therapie für männliche Jugendliche mit Symptomen einer Borderline-Persönlichkeitsstörung. Prax Kinderpsychol Kinderpsychiatr 66(2):104-120

Hinz A (2007) Prävention durch Unterstützung bei der Bewältigung geschlechts- spezifischer Entwicklungsaufgaben. Z Pädagog Psychol 21(2):145-155

Hoffman S, Cummings AL, Leschied AW (2004) Treating aggression in high-risk adolescent girls: A preliminary evaluation. Canad J Couns 38(2):59-74

López-Guimerà G, Sánchez-Carracedo D, Fauquet J, Portell M, Raich RM (2011) Impact of a School-Based Disordered Eating Prevention Program in Adolescent Girls: General and Specific Effects Depending on Adherence to the Interactive Activities. Span J Psychol 14(1):293-303

Lucassen MFG, Merry SN, Hatcher S, Frampton CMA (2015) Rainbow SPARX: A Novel Approach to Addressing Depression in Sexual Minority Youth. Cogn Behav Pract 22(2):203-216

McVey GL, Davis R (2002) A Program to Promote Positive Body Image: A 1-Year Follow-Up Evaluation. J Early Adolesc 22(1):96-108

McVey GL, Davis R, Tweed S, Shaw BF (2004) Evaluation of a school-based program designed to improve body image satisfaction, global self- esteem, and eating attitudes and behaviors: A replication study. Int J Eat Disord 36(1):1-11

O'Callaghan P, McMullen J, Shannon C, Rafferty H, Black A (2013) A randomized controlled trial of trauma-focused cognitive behavioral therapy for sexually exploited, war-affected Congolese girls. J Am Acad Child Adolesc Psychiatry 52(4):359-369

Pepler D, Walsh M, Yuile A, Levene K, Jiang D, Vaughan A et al (2010) Bridging the Gender Gap: Interventions with Aggressive Girls and Their Parents. Prev Sci 11(3):229-238

Raich RM, Sánchez-Carracedo D, López-Guimerà G, Portell M, Moncada A, Fauquet J (2008) A controlled assessment of school-based preventive programs for reducing eating disorder risk factors in adolescent Spanish girls. Eat Disord 16(3):255-272

Roberts-Lewis AC, Welch-Brewer CL, Jackson MS, Pharr OM, Parker S (2010) Female juvenile offenders with HEART: Preliminary findings of an intervention model for female juvenile offenders with substance use problems. J Drug Issues 40(3):611-625

Schinke SP, Schwinn T (2005) Gender-specific computer-based intervention for preventing drug abuse among girls. Am J Drug Alcohol Abuse 31(4):609-616

Schinke SP, Cole KC, Fang L (2009a) Gender-specific intervention to reduce underage drinking among early adolescent girls: a test of a computer-mediated, mother-daughter program. J Stud Alcohol Drugs 70(1):70-77

Schinke SP, Fang L, Cole KC (2009b) Preventing substance use among adolescent girls: 1-year outcomes of a computerized, mother–daughter program. Addict Behav 34(12):1060-1064

Schinke SP, Fang L, Cole KC, Cohen-Cutler S (2011) Preventing substance use among Black and Hispanic adolescent girls: Results from a computer-delivered, mother–daughter intervention approach. Subst Use Misuse 46(1):35-45

Schwinn TM, Schinke SP, Di Noia J (2010) Preventing drug abuse among adolescent girls: outcome data from an internet-based intervention. Prev Sci 11(1):24-32

Schwinn TM, Schinke SP, Hopkins J, Keller B, Liu X (2018) An Online Drug Abuse Prevention Program for Adolescent Girls: Posttest and 1-Year Outcomes. J Youth Adolesc 47(3):490-500

Schwinn TM, Schinke SP, Keller B, Hopkins J (2019) Two-and three-year follow-up from a gender-specific, web-based drug abuse prevention program for adolescent girls. Addict Behav 93:86-92

Schwinn TM, Thom B, Schinke SP, Hopkins J (2015) Preventing drug use among sexual-minority youths: findings from a tailored, web-based intervention. J Adolesc Health 56(5):571-573

Smith DK, Chamberlain P, Deblinger E (2012) Adapting Multidimensional Treatment Foster Care for the Treatment of Co-occurring Trauma and Delinquency in Adolescent Girls. J Child Adolesc Trauma 5(3):224-238

Stewart DA, Carter JC, Drinkwater J, Hainsworth J, Fairburn CG (2001) Modification of eating attitudes and behavior in adolescent girls: A controlled study. Int J Eat Disord 29(2):107-118

Stice E, Marti CN, Spoor S, Presnell K, Shaw H (2008) Dissonance and healthy weight eating disorder prevention programs: long-term effects from a randomized efficacy trial. J Consult Clin Psychol 76(2):329-340

Stice E, Rohde P, Gau J, Shaw H (2009) An effectiveness trial of a dissonance-based eating disorder prevention program for high-risk adolescent girls. J Consult Clin Psychol 77(5):825-834

Stice E, Rohde P, Shaw H, Gau J (2011) An effectiveness trial of a selected dissonance-based eating disorder prevention program for female high school students: Long-term effects. J Consult Clin Psychol 79(4):500-508

Stice E, Shaw H, Burton E, Wade E (2006) Dissonance and healthy weight eating disorder prevention programs: a randomized efficacy trial. J Consult Clin Psychol 74(2):263-275

Suarez E, Jackson DS, Slavin LA, Michels MS, McGeehan KM (2014) Project Kealahou: Improving Hawai ‘i's System of Care for At-Risk Girls and Young Women through Gender-Responsive, Trauma-Informed Care. Hawai'i J Med Public Health 73(12):387-392

Turner LK, Werner-Wilson RJ (2008) Phenomenological experience of girls in a single-sex day treatment group. J Fem Fam Ther 20(3):220-250

Walker SC, Duong M, Hayes C, Berliner L, Leve LD, Atkins DC et al (2019) A tailored cognitive behavioral program for juvenile justice-referred females at risk of substance use and delinquency: A pilot quasi-experimental trial. PloS One 14(11):e0224363

Walsh MM, Pepler DJ, Levene KS (2002) A Model Intervention for Girls with Disruptive Behaviour Disorders: The Earlscourt Girls Connection. Canad J Couns 36(4):297-311

Weigel A, Gumz A, Uhlenbusch N, Wegscheider K, Romer G, Löwe B (2015) Preventing eating disorders with an interactive gender-adapted intervention program in schools: Study protocol of a randomized controlled trial. BMC Psychiatry 15:21

Welch CL, Roberts-Lewis AC, Parker S (2009) Incorporating gender specific approaches for incarcerated female adolescents: Multilevel risk model for practice. J Offender Rehabil 48(1):67-83

Whaling KM, der Sarkissian A, Sharkey J, Akoni LC (2020) Featured counter-trafficking program: Resiliency Interventions for Sexual Exploitation (RISE). Child Abuse Negl 100:104139

Wick K, Brix C, Bormann B, Sowa M, Strauss B, Berger U (2011) Real-world effectiveness of a German school-based intervention for primary prevention of anorexia nervosa in preadolescent girls. Prev Med 52(2):152-158
