# Supplementary material for: Systematic Review of Gender-Specific Child and Adolescent Mental Health Care
Source: Child Psychiatry Hum Dev. 2023 Feb 27;55(6):1487–501. doi: 10.1007/s10578-023-01506-z (PMC11485121; doi:10.1007/s10578-023-01506-z)
Supplement: Supplementary file 3 — Supplementary file3 (DOCX 39 kb) [file 10578_2023_1506_MOESM3_ESM.docx]

**Appendix C:** Excluded studies in full-text screening and reasons for exclusion (*n* = 102)

| **Author(s) and year** | **Title** | **Reason for exclusion** |
| --- | --- | --- |
| Bendall et al., 2020 | A Systematic Review and Synthesis of Trauma-Informed Care Within Outpatient and Counseling Health Settings for Young People | Wrong publication type |
| Brothers et al., 2014 | EVOLUTION—Taking Charge and Growing Stronger: The Design, Acceptability, and Feasibility of a Secondary Prevention Empowerment Intervention for Young Women Living with HIV | Meeting several exclusion criteria |
| Eisenberg et al., 2020 | "It's kind of hard to go to the doctor's office if you're hated there." A call for gender-affirming care from transgender and gender diverse adolescents in the United States | Meeting several exclusion criteria |
| Fortunato et al., 2020 | Caring for gender diverse children and adolescents in Italy: A mixed-method investigation of clinicians' knowledge and approach to clinical practice | No specific intervention |
| Friedrich et al., 2010 | Gender as a Factor in School-Based Mental Health Service Delivery | Wrong publication type |
| Hendricks, & Testa, 2012 | A Conceptual Framework for Clinical Work With Transgender and Gender Nonconforming Clients: An Adaptation of the Minority Stress Model | Meeting several exclusion criteria |
| Hubbard, & Matthews, 2008 | Reconciling the differences between the "Gender-Responsive" and the "What Works" literatures to improve services for girls | Wrong publication type |
| Javdani, & Allen, 2016 | An Ecological Model for Intervention for Juvenile Justice-Involved Girls: Development and Preliminary Prospective Evaluation | Focus not on mental health |
| Kaslow, & Aronson, 2004 | Recommendations for family interventions following a suicide | No specific intervention |
| Kiss et al., 2020 | Male and LGBT survivors of sexual violence in conflict situations: a realist review of health interventions in low-and middle-income countries | Wrong publication type |
| Leve, & Chamberlain, 2007 | A Randomized evaluation of multidimensional treatment foster care: Effects on school attendance and homework completion in juvenile justice girls | Focus not on mental health |
| Matthews, & Hubbard, 2008 | Moving ahead: Five essential elements for working effectively with girls | Meeting several exclusion criteria |
| Ravoira et al., 2012 | Urgent Work: Developing a Gender-Responsive Approach for Girls in the Juvenile Justice System | Meeting several exclusion criteria |
| Shilo et al., 2021 | Gender Differences among Commercially Sexually Exploited Youth in Israel | Adult population |
| Staller, & Faraone, 2006 | Attention-deficit hyperactivity disorder in girls - Epidemiology and management | Intervention not gender-specific |
| Ulrich et al., 2021 | Explaining psychosocial care among unaccompanied minor refugees: a realist review | Meeting several exclusion criteria |
| Van Damme et al., 2017 | The Good Lives Model among detained female adolescents | Meeting several exclusion criteria |
| Van Vugt et al., 2016 | Can Institutionalized Adolescent Females With a Substantiated History of Sexual Abuse Benefit From Cognitive Behavioral Treatment Targeting Disruptive and Delinquent Behaviors? | Intervention not gender-specific |
| Warin, & Price, 2020 | Transgender awareness in early years education (EYE): 'we haven't got any of those here' | Meeting several exclusion criteria |
| Ziegenhain et al., 2003 | Frühe Elternschaft: jugendliche Mütter und ihre Kinder | Focus not on mental health |
| Bell et al., 2013 | Adolescent and young adult male health: a review | Meeting several exclusion criteria |
| Böge et al., 2020 | Mental health in refugees and asylum seekers (MEHIRA): study design and methodology of a prospective multicentre randomized controlled trail investigating the effects of a stepped and collaborative care model | Meeting several exclusion criteria |
| Chandra et al., 2012 | Schizophrenia in women and children: a selective review of literature from developing countries | Meeting several exclusion criteria |
| Cibich, & Wade, 2019 | Treating bulimia nervosa in the context of gender dysphoria using 10-session cognitive behavior therapy | Meeting several exclusion criteria |
| Foxcroft, & Tsertsvadze, 2011 | Universal family-based prevention programs for alcohol misuse in young people | Meeting several exclusion criteria |
| Golan et al., 2014 | Gender related differences in response to ""in favor of myself"" wellness program to enhance positive self & body image among adolescents | Intervention not gender-specific |
| Granski et al., 2020 | A Meta-Analysis of Program Characteristics for Youth with Disruptive Behavior Problems: The Moderating Role of Program Format and Youth Gender | Wrong publication type |
| Guthrie, & Low, 2000 | A substance use prevention framework: considering the social context for African American girls | No specific intervention |
| Biegler-Vitek, & Schorn, 2013 | „Die Eule liegt zwischen mir und dem Rudi und berührt jeden von uns mit einem Flügel …“Anwendung und Bedeutung der Soziometrie in der psychodrama-therapeutischen Arbeit mit Kindern und Jugendlichen | Not peer-reviewed |
| Judd et al., 2009 | Gender-sensitive mental health care | Meeting several exclusion criteria |
| Gsell, & Binswanger, 2012 | Psychosexuelle Entwicklung und Geschlechtsidentität unter intersexuellen Konditionen | Meeting several exclusion criteria |
| Kumpfer et al., 2008 | A wakeup call to the prevention field: are prevention programs for substance use effective for girls? | Wrong publication type |
| LaBrie et al., 2009 | A brief live interactive normative group intervention using wireless keypads to reduce drinking and alcohol consequences in college student athletes | Intervention not gender-specific |
| Leve et al., 2015 | Risks, Outcomes, and Evidence-Based Interventions for Girls in the US Juvenile Justice System | Wrong publication type |
| Joormann, & Ehlert, 2012 | Mütter und Töchter - eine Schicksalsgemeinschaft? Ein Interview mit Jutta Joormann. | No specific intervention |
| Mahalik et al., 2012 | Developing a taxonomy of helpful and harmful practices for clinical work with boys and men | No specific intervention |
| Malone, 2007 | The impact of peer death on adolescent girls: a task-oriented group intervention | No specific intervention |
| Merry et al., 2004 | Psychological and/or educational interventions for the prevention of depression in children and adolescents | Wrong publication type |
| Morrison-Beedy, & Mazurek Melnyk, 2019 | Making a Case for Integrating Evidence-Based Sexual Risk Reduction and Mental Health Interventions for Adolescent Girls | Intervention not gender-specific |
| Moynihan et al., 2018 | Interventions that Foster Healing Among Sexually Exploited Children and Adolescents: A Systematic Review | Wrong publication type |
| Wahl et al., 2011 | A school-based universal programme to prevent depression and to build up life skills | Intervention not gender-specific |
| Peate, 2010 | The mental health of men and boys: an overview | No specific intervention |
| Purtle et al., 2016 | Gender Differences in Posttraumatic Stress Symptoms Among Participants of a Violence Intervention Program at a Pediatric Hospital: A Pilot Study | Intervention not gender-specific |
| Quinn, 2005 | Treating adolescent girls and women with ADHD: gender-specific issues | Wrong publication type |
| Rucklidge, 2008 | Gender differences in ADHD: implications for psychosocial treatments | Intervention not gender-specific |
| Shalanski, & Ewashen, 2019 | An interpretive phenomenological study of recovering from mental illness: Teenage girls' portrayals of resilience | No specific intervention |
| Simons et al., 2014 | Understanding gender variance in children and adolescents | Meeting several exclusion criteria |
| Strömbäck et al., 2013 | Girls need to strengthen each other as a group': experiences from a gender-sensitive stress management intervention by youth-friendly Swedish health services--a qualitative study | Adult population |
| Strömbäck et al., 2016 | Gender-sensitive and youth-friendly physiotherapy: Steps toward a stress management intervention for girls and young women | Adult population |
| Thomann et al., 2020 | Intervention Response to the Trauma-Exposed, Justice-Involved Female Youth: A Narrative Review of Effectiveness in Reducing Recidivism | Focus not on mental health |
| Van Donge et al., 2019 | Transgender Dependent Adolescents in the U.S. Military Health Care System: Demographics, Treatments Sought, and Health Care Service Utilization | Meeting several exclusion criteria |
| Watkins et al., 2017 | Strengths and weaknesses of the Young Black Men, Masculinities, and Mental Health (YBMen) Facebook project | Adult population |
| Zenker, 2005 | Sucht und Geschlecht | No specific intervention |
| Moretti et al., 2004 | Girls and aggression: Contributing factors and intervention principles | Not peer-reviewed |
| Babinski et al., 2013 | The effects of single versus mixed gender treatment for adolescent girls with ADHD | Intervention not gender-specific |
| Barrow, & Apostle, 2018 | Addressing mental health conditions often experienced by transgender and gender expansive children | Meeting several exclusion criteria |
| Baumann et al., 2009 | Cognitive behavioral programming for women and girls | Not peer-reviewed |
| Benjet, 2009 | Gender sensitive psychiatric care for children and adolescents | Not peer-reviewed |
| Berger et al., 2014 | Effectiveness of reducing the risk of eating-related problems using the German school-based intervention program, 'Torera', for preadolescent boys and girls | Intervention not gender-specific |
| Brooks, 2001 | Developing gender awareness: When therapist growth promotes family growth | Wrong publication type |
| Cauffman, 2008 | Understanding the female offender | Meeting several exclusion criteria |
| Chamberlain, & Moore, 2002 | Chaos and trauma in the lives of adolescent females with antisocial behavior and delinquency | Focus not on mental health |
| Dodds et al., 2003 | Retention, adherence, and compliance: Special needs of HIV-infected adolescent girls and young women | Adult population |
| Ellis et al., 2000 | Profile-based intervention: Developing gender-sensitive treatment for adolescent substance abusers | Intervention not gender-specific |
| Emerson et al., 2001 | Using creative arts to build coping skills to reduce domestic violence in the lives of female juvenile offenders | Focus not on mental health |
| Feder et al., 2010 | Boys and violence: A gender-informed analysis | Wrong publication type |
| Freitag, 2014 | Neurobiology and treatment of adolescent female conduct disorder: FemNAT-CD consortium: A new European cooperation | Wrong publication type |
| Gillham, & Chaplin, 2011 | Preventing girls' depression during the transition to adolescence | Not peer-reviewed |
| Griffith et al., 2001 | Family interventions for depressed African American adolescent females | Not peer-reviewed |
| Trial Registr., 2017 (Related article: Tanksale et al., 2021) | Evaluating the effectiveness of mind-body skills training based on yoga techniques in children on the Autism Spectrum | Intervention not gender-specific |
| Trial Registr., 2018 (Related article: Hides et al., 2020) | Keep it Real: a web-based program for psychotic-like experiences and cannabis use | Meeting several exclusion criteria |
| Chamberlain et al., 2007 | Multidimensional treatment foster care for girls in the juvenile justice system: 2-year follow-up of a randomized clinical trial | Focus not on mental health |
| Irvine et al., 2017 | Lesbian, bisexual, questioning, gender-nonconforming, and transgender (LBQ/GNCT) girls in the juvenile justice system: Using an intersectional lens to develop gender-responsive programming | Meeting several exclusion criteria |
| Karim, 2012 | Adolescent behaviors out of control: An introduction to adolescent residential treatment | Meeting several exclusion criteria |
| Kelly et al., 2006 | Girl World: A Primary Prevention Program for Mexican American Girls | Focus not on mental health |
| Kessler et al., 2007 | Innovative problem-solving court models for justice-involved youth | Not peer-reviewed |
| Kipnis, 2004 | Gender, sex, and professional ethics in child and adolescent psychiatry | Meeting several exclusion criteria |
| Lanctot, 2018 | Gender-responsive programs and services for girls in residential centers: Meeting different profiles of rehabilitation needs | Meeting several exclusion criteria |
| Daley & Lecroy, 2001 | Empowering adolescent girls: Examining the present and building skills for the future with The Go Grrrls Program | Not peer-reviewed |
| Leibowitz et al., 2019 | Walking a tightrope: A child and adolescent psychiatry perspective on the spectrum of affirmation and pathologization with gender diverse youth | Not peer-reviewed |
| Leibowitz, & de Vries, 2016 | Gender dysphoria in adolescence | No specific intervention |
| Trial Registr., 2018 (Related article: Ho et al., 2019) | Promoting Smoking Cessation for Female Smokers in Hong Kong Through Training Female Youth SCRA | Adult population |
| Pepler et al., 2004 | Interventions for aggressive girls: Tailoring and measuring the fit | Not peer-reviewed |
| Rice, 2015 | Violence among young men: The importance of a gender-specific developmental approach to adolescent male suicide and homicide | Meeting several exclusion criteria |
| Sandberg, & Mazur, 2014 | A noncategorical approach to the psychosocial care of persons with DSD and their families | Meeting several exclusion criteria |
| Schwartz, 2012 | Listening to children imagining gender: Observing the inflation of an idea | No specific intervention |
| Sharon, 2016 | Psychiatry's role in supporting healthy development in gender diverse children | No specific intervention |
| Stanard, 2000 | Assessment and treatment of adolescent depression and suicidality | Intervention not gender-specific |
| Stoddard et al., 2011 | Improving medical education about gender-variant youth and transgender adolescents | No specific intervention |
| Künzel, & Metzner, 2011 | Mädchen in der Kindermusiktherapie. Eine Literaturrecherche zu Gender-Aspekten | No specific intervention |
| Preißmann, 2013 | Mädchen und Frauen mit Autismus-Spektrum-Störung | No specific intervention |
| Green et al., 2019 | Women and Autism Spectrum Disorder: Diagnosis and Implications for Treatment of Adolescents and Adults | Intervention not gender-specific |
| Arndt, & Aschermann, 2005 | Geschlechtsspezifische Behandlungswünsche von Patientinnen und Patienten in der Kinder- und Jugendpsychiatrie | Meeting several exclusion criteria |
| Barth, 2012 | "Der Weg zum Mann ist auch nicht leicht" – Essstörungen bei Jungen | Not peer-reviewed |
| Behn at al., 2008 | Themenschwerpunkt Anti-Gewalt-Trainings und Genderorientierung | Not peer-reviewed |
| Gahleitner et al., 2008 | Neue Bindungen wagen. Beziehungsorientierte Arbeit mit traumatisierten Mädchen in der Einrichtung "Myrrha" | Not peer-reviewed |
| Junglas, 2005 | Geschlechtergerechte Psychotherapie und Psychiatrie. "Zwei Seelen wohnen, ach! In meiner Brust" | Not peer-reviewed |
| Kottmann, & Zeller, 2012 | "Wer bin ich? - Ich bin wer!"". Gesundheitsförderung und Prävention von Essstörungen mit Jugendlichen | Not peer-reviewed |
| Rohde et al., 2006 | Geschlechtsspezifische Psychiatrie und Psychotherapie. Ein Handbuch | Not peer-reviewed |
| Burke, & Loeber, 2015 | The effectiveness of the Stop Now and Plan (SNAP) program for boys at risk for violence and delinquency | Intervention not gender-specific |
| Thomasius et al., 2009 | Herausforderungen und Perspektiven in der Jugend-Suchthilfe | Meeting several exclusion criteria |
| Wiegand-Grefe et al., 2011 | Kinder und ihre psychisch kranken Eltern. Familienorientierte Prävention - der CHIMPs-Beratungsansatz | Intervention not gender-specific |

*Note.* Reviews were not included, but screened for relevant articles, if they met the other inclusion criteria (e.g. mental health interventions for children and adolescents).
